# Albuminuria Responses to Dapagliflozin in Patients With Type 2 Diabetes: A Crossover Trial

**DOI:** 10.1001/jamanetworkopen.2025.1689

**Published:** 2025-03-24

**Authors:** Jelle M. Beernink, Niels Jongs, Cees J. A. Doelman, Gozewijn D. Laverman, Hiddo J. L. Heerspink

**Affiliations:** 1 Department of Clinical Pharmacy and Pharmacology, University Medical Center Groningen, Groningen, the Netherlands; 2 Department of Internal Medicine, Ziekenhuis Groep Twente, Almelo, the Netherlands; 3 Department of Clinical Chemistry, Medlon Laboratory Diagnostics, Unilabs, Enschede, the Netherlands; 4 Biomedical Signals and Systems, University of Twente, Enschede, the Netherlands

## Abstract

**Importance:**

Dapagliflozin reduces the urine albumin-to-creatinine ratio (UACR) and estimated glomerular filtration rate (eGFR) decline at a population level, but individuals show a large variation in responses. The n-of-1 trial design allows for direct assessment of treatment effects within an individual, and digital technologies and remote study assessments can reduce clinic visits, ease participant burden, and improve trial efficiency.

**Objective:**

To assess individual UACR responses to dapagliflozin treatment in a decentralized clinical trial and the feasibility of remote data collection.

**Design, Setting, and Participants:**

This decentralized, randomized, double-blind, placebo-controlled crossover trial using an n-of-1 approach was conducted using data from the Dutch primary and secondary health care systems between May 2021 and September 2022. Participants included adults with type 2 diabetes, a UACR greater than 20 mg/g, and an eGFR greater than 30 mL/min/1.73 m^2^. Statistical analyses were performed between June and August 2023.

**Interventions:**

Participants were assigned to two 1-week treatment periods with dapagliflozin, 10 mg/d, and two 1-week treatment periods with placebo in random order, with 1-week washout periods in between.

**Main Outcomes and Measures:**

The primary outcome was the difference in the change in UACR from start to end of treatment between dapagliflozin and placebo in the per-protocol population. A post hoc exploratory analysis assessed the feasibility of remote data collection, including the proportion of urine and capillary blood samples successfully delivered to the central laboratory.

**Results:**

In total, 20 participants (mean [SD] age, 64.9 [8.7] years; 17 [85.0%] male) with a mean (SD) eGFR of 70.2 (20.3) mL/min/1.73 m^2^ and a median UACR of 94.7 (IQR, 29.8-242.6) mg/g were included in the study. They experienced a relative change in UACR with dapagliflozin compared with placebo of –15.1% (95% CI, –28.2% to –3.3%; *P* = .01). UACR changes showed considerable variation during both dapagliflozin and placebo treatment (first treatment period: median, –12.8% [range, –56.3% to 36.2%] and 2.9% [range, –86.7% to 35.1%], respectively). UACR changes correlated significantly between the first and second dapagliflozin exposure (*r* = 0.50; *P* = .03), with no correlation observed between the placebo exposure periods (*r* = 0.09; *P* = .69). With regard to remote data collection, 811 of 816 urine samples (99.4%) and 433 of 440 capillary blood samples (98.4%) were successfully delivered to the central laboratory.

**Conclusions and Relevance:**

In this crossover trial, individual UACR responses to dapagliflozin reflected a pharmacological response. Remote data collection proved to be reliable, supporting its use in future studies and clinical practice for monitoring individual dapagliflozin responses.

**Trial Registration:**

EudraCT identifier: 2020-004929-23

## Key Points

**Questions:**

What is the individual response to dapagliflozin treatment among patients with albuminuria, and is it consistent after re-exposure?

**Findings:**

In this crossover trial of 20 adults with type 2 diabetes and albuminuria, dapagliflozin treatment reduced albuminuria by 15.1% compared with placebo, with considerable between-individual variation. The albuminuria response was consistent after dapagliflozin re-exposure.

**Meaning:**

The individual albuminuria response to dapagliflozin reflected a pharmacological effect.

## Teaser

This crossover trial compares individual urine albumin-creatinine ratio responses to dapagliflozin therapy, after re-exposure to dapagliflozin, and to placebo among patients with type 2 diabetes and albuminuria.

## Introduction

Albuminuria is an established risk marker for progression of chronic kidney disease (CKD) and cardiovascular disease in patients with or without type 2 diabetes.^1^ Dapagliflozin, a selective sodium-glucose cotransporter 2 (SGLT2) inhibitor, slows estimated glomerular filtration rate (eGFR) decline, reduces the risks of kidney failure and cardiovascular outcomes, and improves risk factors of CKD progression, including urine albumin-to-creatinine ratio (UACR), glycated hemoglobin (HbA_1c_) level, and blood pressure.^2,3,4,5,6,7,8,9^ While these benefits are evident at a population level, individual responses to dapagliflozin in CKD risk markers and eGFR decline vary significantly.^2,10^ Whether this individual response heterogeneity is a pharmacological response variation or random measurement variation cannot be established in traditional parallel-group clinical trials. N-of-1 trials, in which participants are exposed to both active and placebo interventions in random order, provide more precise assessments of drug efficacy and safety at the individual level.^11^

In conventional clinical trials, frequent onsite visits for health assessments and biomaterial collections can deter some patients, hindering recruitment and increasing participant burden. Digital technologies and wearable devices allow remote assessments (ie, while participants are at home), reducing clinic visits, decreasing participant time commitment, alleviating participant burden, and streamlining clinical trial operations.^12^

We conducted a decentralized crossover randomized trial involving repeated administration (ie, a series of n-of-1 trials) to assess individual UACR responses to dapagliflozin compared with placebo. We hypothesized that UACR changes with dapagliflozin would be consistent after re-exposure and that remote urine collection would be a feasible approach for testing individual UACR responses.

## Methods

### Study Design and Participants

This decentralized, randomized, placebo-controlled, double-blind crossover trial using an n-of-1 approach was conducted with participants recruited from the outpatient clinic of the Department of Internal Medicine at Hospital Group Twente in Almelo, the Netherlands, and from general practitioner practices. Eligible participants had type 2 diabetes, were aged 18 years or older, had a UACR greater than 20 mg/g (2.26 mg/mmol), had an eGFR greater than 30 mL/min/1.73 m^2^, and were receiving a stable dose of an angiotensin-converting enzyme inhibitor or angiotensin receptor blocker unless not tolerated. Key exclusion criteria included a diagnosis of type 1 diabetes, prior SGLT2 inhibitor use within 4 weeks before randomization, or inability to monitor physiological parameters or use digital technologies. Ethical approval was obtained from the Medical Research Ethics Committee of University Medical Center Groningen, and the trial adhered to the International Conference on Harmonization Good Clinical Practice Guidelines and the Declaration of Helsinki.^13^ All participants provided written informed consent. The full trial protocol and statistical analysis plan are provided in Supplement 1. The Consolidated Standards of Reporting Trials (CONSORT) reporting guideline was followed.^14^

### Randomization and Masking

Participants were randomized to a crossover trial with two 1-week treatment periods with dapagliflozin, 10 mg/d, and two 1-week treatment periods with placebo in random order, with 1-week washout periods between treatment periods to prevent carryover effects. The following 4 treatment sequences were used: (1) placebo, dapagliflozin, placebo, dapagliflozin (n = 6); (2) placebo, dapagliflozin, dapagliflozin, placebo (n = 5); (3) dapagliflozin, placebo, dapagliflozin, placebo (n = 4); and (4) dapagliflozin, placebo, placebo, dapagliflozin (n = 5). Randomization was performed using a computer-generated random sequence and a validated interactive web-response system by an independent pharmacy staff member. All participants, investigators, and the sponsor were blinded to the treatment assignment. The placebo and active tablets were identical in color, shape, size, texture, and taste.

### Procedures

The trial was conducted remotely in the participants’ home environments. Participants collected all data using wearable and digital devices. Measurements were taken on prespecified days. The initial clinic visit, held within 2 weeks before the 1-week run-in period, served as a screening in which participants underwent eligibility assessments and had eGFR, HbA_1c_, and UACR measurements. Self-reported race and ethnicity were recorded to characterize the diversity of the study population. Participants could choose from the following specific race and ethnicity categories: African, Asian, Latino, White, multiple races, and other race. After eligibility was confirmed, participants started a 1-week run-in period following receipt of the instructions for using digital technologies and collecting urine and capillary blood samples. Telephone calls were conducted on days 3 and 6 of the first 2 treatment periods to assess adherence to study procedures and the occurrence of adverse events. In addition, participants could contact the study investigators when needed.

Participants collected first-morning void urine samples using the PeeSpot device (Hessels+Grob) once daily on 40 separate days. This device is a validated tool for collecting and preserving small urine samples.^15^ After collection, samples were stored in a refrigerator and sent to the central laboratory for UACR measurement. To enhance precision and account for day-to-day variation in UACR, participants collected urine samples on 3 consecutive days during each 1-week trial period.

Participants collected capillary blood samples on 22 separate days using a BD-Microtainer Contact-Activated Lancet (Franklin Lakes) and Hem-Col tubes (Labonovum). Samples were collected on the first day and the last 2 days of each treatment period as well as on the last 2 days of each washout period. Collection tubes were mailed to the central laboratory for creatinine measurement. A venous blood sample was collected at a second study visit to compare clinical chemistry results between capillary and venous blood samples. Capillary blood samples were also used to determine fasting plasma glucose level using either an eligible personal glucose meter or an Accu-Chek Instant System (Roche Diagnostics).

Participants measured blood pressure using a Withings BPM Connect once daily on 28 separate days, with 3 consecutive readings per session. Body weight was measured using the Withings Body Smart scale once daily on 40 separate days. Blood pressure and body weight were recorded on 3 consecutive days during each 1-week trial period. Participants connected the devices to the Withings mobile app, which was linked to a secure electronic data capture environment (Selfcare BV). This setup enabled automatic data transfer from the devices to the app and subsequently to the electronic data capture environment, eliminating the need for manual data entry or uploads. At the end of the study, blood pressure and body weight data were exported from the electronic data capture environment to the final analysis database.

Medication was dispensed in bottles with Medication Electronic Monitoring System caps (AARDEX Group), which recorded the exact data and time each bottle was opened, allowing real-time monitoring of adherence. The data were transmitted via a reader to adherence software for storage and analysis.

A self-designed questionnaire created specifically for this study and not based on validated instruments was used to assess participant experience and satisfaction (eMethods in Supplement 2; translated from Dutch to English). It consisted of 6 multiple choice and 2 open-ended questions. Multiple choice questions used a 5-point Likert scale (from very difficult to very easy) to evaluate the ease of remote data collection. The questionnaire aimed to gather insights to guide and inform the design of future trials and clinical practice.

Participants were instructed to take the study medication once daily in the morning. The dosing regimen was based on prior research showing that the effects of dapagliflozin on UACR, blood pressure, body weight, and eGFR were fully present after 1 week of treatment and returned to baseline about 4 days after stopping treatment.^16^ Therefore, 1-week treatment periods with 1-week washouts were deemed sufficient to assess treatment effects.

### Outcomes

The primary outcome was the difference in UACR change from start to end of treatment between dapagliflozin and placebo in the per-protocol population. Secondary outcomes were changes in systolic blood pressure, body weight, eGFR, and fasting plasma glucose level. A post hoc exploratory outcome assessed the feasibility of remote data collection, defined as the proportion of successful laboratory deliveries of urine and capillary blood samples, the proportion of recorded blood pressure and body weight readings in the electronic data capture environment, questionnaire responses, and medication adherence. The correlation between creatinine values obtained from capillary and venous blood samples was another post hoc exploratory outcome.

### Statistical Analysis

The sample size was based on the hypothesis that dapagliflozin responses are reproducible after re-exposure, showing a correlation between the first and second exposures, with no correlation expected during placebo. A prior study showed albuminuria response correlation coefficients of 0.70 to 0.90 during renin-angiotensin system intervention.^17^ However, given the retrospective nature of that study, a more conservative correlation coefficient of 0.60 was adopted for the current study. A sample size of 20 ensured higher than 80% power (at α = .05) to detect a correlation coefficient of 0.60.

Due to the skewed distribution, UACR values were log transformed before analysis. Baseline values were calculated as the geometric mean of urine samples collected on days –3, –2, and –1 during the run-in period and days 5, 6, and 7 during each washout period. End-of-treatment values were calculated as the geometric mean of urine samples collected on days 5, 6, and 7 of each active treatment period. Similar to other trials, the ratio of the end-of-treatment to baseline UACR values was used to calculate the relative change from baseline.

The study included 2 cycles for each treatment period (dapagliflozin and placebo). UACR differences between dapagliflozin and placebo were assessed using a linear mixed-effects model with random intercepts and slopes for treatment. Fixed effects included treatment sequence and cycle. Appropriate contrasts were implemented to assess the placebo-subtracted response and correlations across cycles. The variance-covariance pattern of the mixed-effects model was assumed to be unstructured. Secondary outcomes were analyzed like the primary outcome but without log transformation. Changes in blood pressure, body weight, eGFR, and fasting plasma glucose level were based on the means from days 6 and 7 during active and washout periods.

All statistical analyses were performed using R, version 4.10 (R Project for Statistical Computing). Statistical analyses were performed between June and August 2023. A 2-sided *P* < .05 was considered statistically significant.

## Results

**Figure 1.**
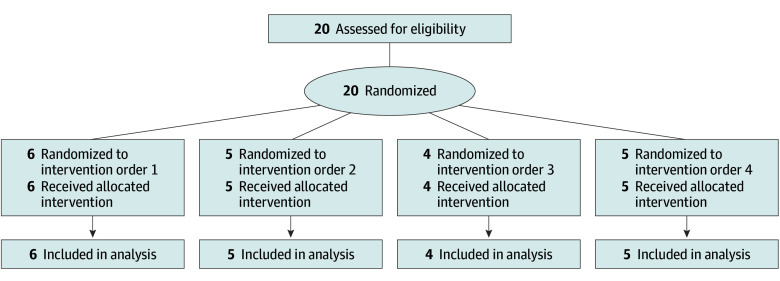
Flow Diagram of Study Participants Order 1 was placebo, dapagliflozin, placebo, and dapagliflozin; order 2, placebo, dapagliflozin, dapagliflozin, and placebo; order 3, dapagliflozin, placebo, dapagliflozin, and placebo; and order 4, dapagliflozin, placebo, placebo, and dapagliflozin.

Between May 11, 2021, and September 13, 2022, 20 participants were screened, all of whom completed the study and were assessed for the primary and secondary study outcomes (Figure 1). No participants discontinued the study or medication. The mean (SD) age was 64.9 (8.7) years, with 3 (15.0%) female and 17 (85.0%) male participants; all were White. The median UACR was 94.7 (IQR, 29.8-242.6) mg/g, mean (SD) eGFR was 70.2 (20.3) mL/min/1.73 m^2^, mean (SD) body weight was 100.3 (18.3) kg, mean (SD) body mass index (calculated as weight in kilograms divided by height in meters squared) was 31.3 (5.0), and mean (SD) systolic blood pressure was 142.3 (15.3) mm Hg (Table). The median diabetes duration was 10.8 (IQR, 4.8-19.2) years. The mean (SD) HbA_1c_ percentage of total hemoglobin was 7.5% (3.0%) (to convert to proportion of total hemoglobin, multiply by 0.01), and the mean (SD) HbA_1c_ level was 58.2 (9.5) mmol/mol. In total, 19 participants (95.0%) were receiving an angiotensin-converting enzyme inhibitor or angiotensin receptor blocker.

**Table.** Baseline Characteristics

Characteristic	Participants, No. (%)
Age, mean (SD), y	64.9 (8.7)
Sex	
Female	3 (15.0)
Male	17 (85.0)
Race	
White	20 (100)
Diabetes duration, median (IQR), y	10.8 (4.8-19.2)
Body weight, mean (SD), kg	100.3 (18.3)
BMI, mean (SD)	31.3 (5.0)
BMI distribution	
<25	3 (15.0)
≥25 to <30	5 (25.0)
≥30 to <35	8 (40.0)
≥35 to <40	4 (20.0)
HbA_1c_ level, mean (SD), %	7.5 (3.0)
HbA_1c_ level, mean (SD), mmol/mol	58.2 (9.5)
UACR, median (IQR), mg/g	94.7 (29.8-242.6)
UACR, median (IQR), mg/mmol	10.7 (3.4-27.4)
Blood pressure, mean (SD), mmHg	
Systolic	142.3 (15.3)
Diastolic	86.4 (10.6)
eGFR, mean (SD), mL/min/1.73 m^2^	70.2 (20.3)
Concomitant medication	
ACE inhibitor	6 (30.0)
ARB	13 (65.0)
Insulin	5 (25.0)
Metformin	15 (75.0)
GLP-1 receptor agonists	7 (35.0)
Cardiovascular history	9 (45.0)

Abbreviations: ACE, angiotensin-converting enzyme; ARB, angiotensin receptor blocker; BMI, body mass index (calculated as weight in kilograms divided by height in meters squared); eGFR, estimated glomerular filtration rate; GLP-1, glucagon-like peptide-1; HbA_1c_, glycated hemoglobin; UACR, urine albumin-to-creatinine ratio.

SI conversion factor: To convert percentage of total HbA_1c_ to proportion of total hemoglobin, multiply by 0.01.

**Figure 2.**
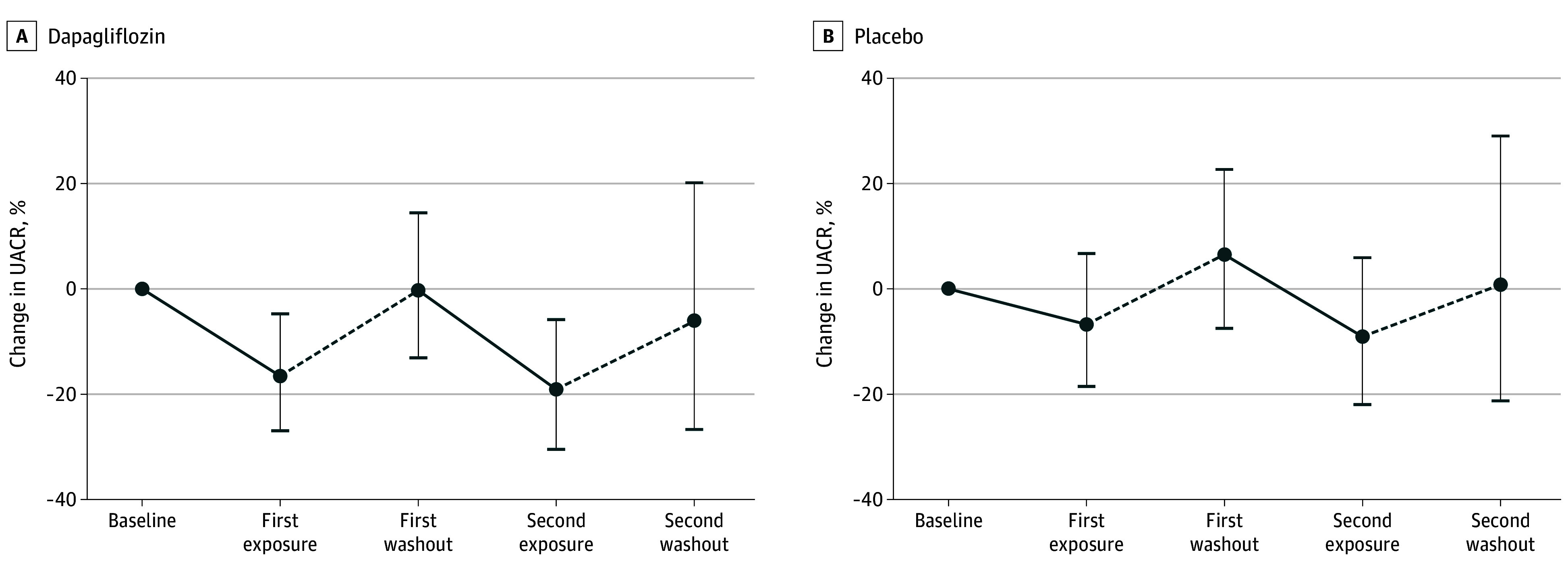
Changes in Urine Albumin-to-Creatinine Ratio (UACR) During Treatment With Dapagliflozin and Placebo Error bars indicate 95% CIs.

Dapagliflozin significantly reduced UACR after both exposures (first exposure: –16.6% [95% CI, –26.9% to –4.7%; second exposure: –19.1% [95% CI, –30.5% to –5.8%]), with UACR returning to baseline during washouts (Figure 2). Placebo showed no significant UACR changes (first exposure: –6.7% [95% CI, –18.5% to 6.7%]; second exposure: –9.1% [95% CI, –21.9% to 6.0%]). Overall, dapagliflozin reduced albuminuria by 15.1% (95% CI, –28.2% to –3.3%; *P* = .01) compared with placebo.

**Figure 3.**
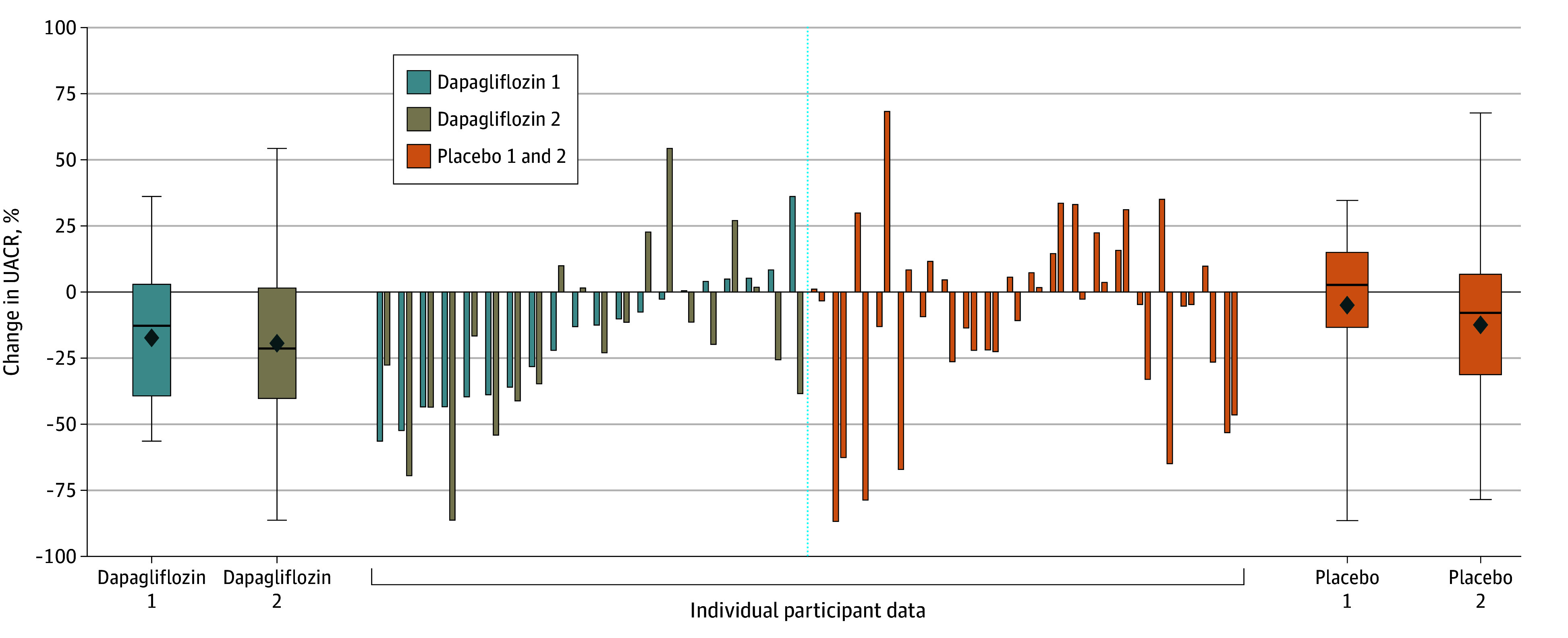
Mean Change in Urine Albumin-to-Creatinine Ratio (UACR) and Individual Participant UACR Changes During Dapagliflozin and Placebo Treatment Periods Box plots demonstrate median (horizontal line in middle of box), mean (diamond), IQR (box top and bottom), and maximum and minimum UACR changes (whiskers).

Individual UACR changes during dapagliflozin and placebo administration varied markedly between individuals (Figure 3). During dapagliflozin treatment, UACR ranged from –56.3% to 36.2% (median, –12.8%) in the first period and –86.2% to 54.4% (median, –21.3%) in the second. For placebo, UACR ranged from –86.7% to 35.1% (median, 2.9%) and –78.6% to 68.3% (median, –7.7%), respectively for the 2 periods.

**Figure 4.**
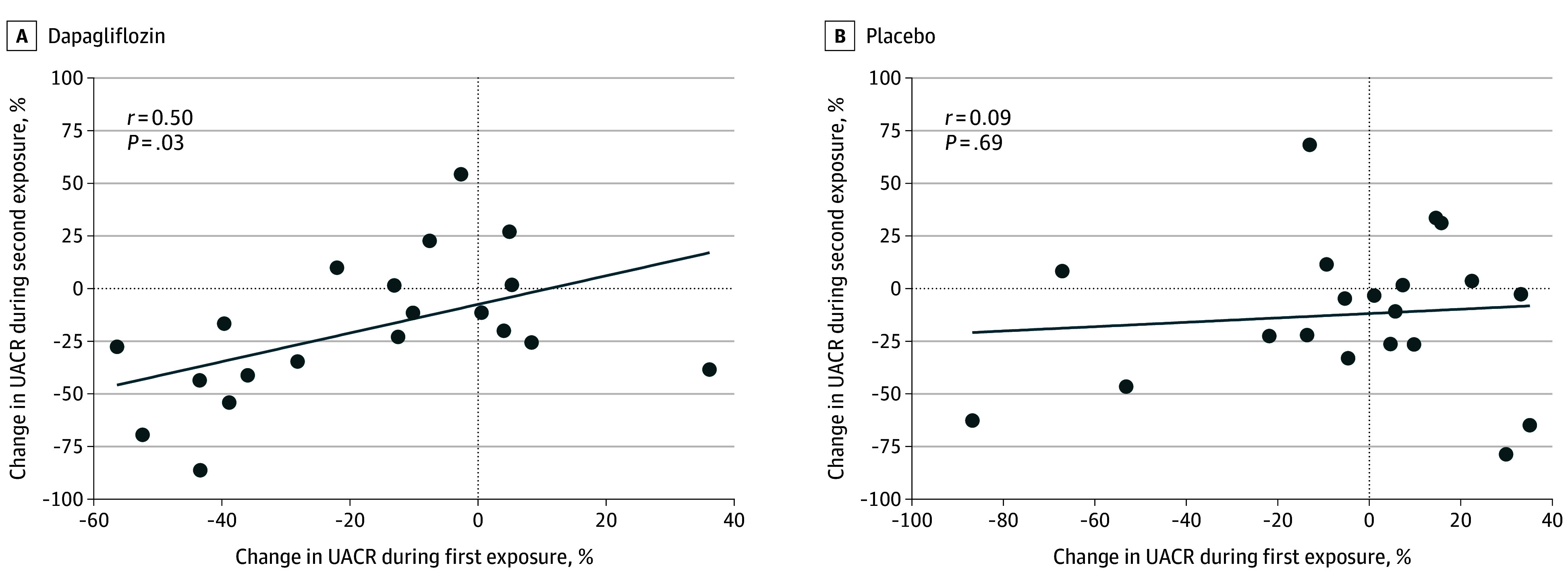
Correlation in Percentage Urine Albumin-to-Creatinine Ratio (UACR) Changes During First and Second Exposure to Dapagliflozin and Placebo

A significant correlation in individual UACR changes was observed between the first and second dapagliflozin period (*r* = 0.50; *P* = .03) (Figure 4A), but not for placebo (*r* = 0.09; *P* = .69) (Figure 4B). When a single urine sample from day 7 of the active treatment periods was used to assess UACR change rather than the geometric mean of 3 urine samples, the individual UACR correlation between the first and second dapagliflozin treatment period was attenuated (*r* = 0.32; *P* = .17).

Compared with placebo, dapagliflozin reduced systolic blood pressure by –5.11 (95% CI, –8.75 to –1.47) mm Hg (*P* = .008) (eFigure 1 in Supplement 2). Dapagliflozin also reduced body weight compared with placebo (–0.53 [95% CI, –0.81 to –0.26] kg; *P* < .001) (eFigure 1 in Supplement 2). Dapagliflozin reduced eGFR during the first and second exposure periods by –10.18 (95% CI, –14.63 to –5.73) mL/min/1.73 m^2^ and –5.69 (95% CI, –11.18 to –0.20) mL/min/1.73 m^2^, respectively (eFigure 1 in Supplement 2). Changes in eGFR during dapagliflozin were reversed during the washout periods. Overall, dapagliflozin reduced eGFR by –5.33 (95% CI, –10.12 to –0.54) mL/min/1.73 m^2^ (*P* = .03) compared with placebo. Dapagliflozin reduced fasting plasma glucose level by –9.03 (95% CI, –17.67 to –0.38) mg/dL (to convert to mmol/L, multiply by 0.0555) compared with placebo.

For remote data collection, 811 of 816 urine samples (99.4%) and 433 of 440 capillary blood samples (98.4%) were successfully delivered to the central laboratory, with 406 of 433 blood samples (93.8%) containing sufficient volume for analysis. Of all scheduled blood pressure and body weight assessments, 545 of 572 (95.3%) and 776 of 808 (96.0%) were successfully completed, respectively. Participants rated remote collection of urine samples (4.45), blood pressure (4.55), and body weight (4.70) as easy on a 5-point Likert scale, while capillary blood sample collection was rated as more difficult (3.05). Installing mobile apps and connecting devices were rated relatively easy (3.93 and 3.86). Medication adherence data were available for 19 of 20 participants (95.0%) over 532 treatment days, with opened bottles on 518 days (97.4%) (eFigure 2 in Supplement 2). Capillary and venous blood creatinine measurements showed a very strong positive correlation (*r* = 0.94; *P* < .001).

Dapagliflozin was generally well tolerated. There were no serious adverse events, and no participant discontinued dapagliflozin treatment due to adverse events after treatment initiation.

## Discussion

We conducted a randomized, placebo-controlled, double-blind crossover trial using an n-of-1 approach that involved 20 patients with type 2 diabetes to assess individual responses to the SGLT2 inhibitor dapagliflozin. Dapagliflozin reduced UACR compared with placebo, although considerable variability was observed among individuals. The UACR changes were consistent across the first and second dapagliflozin treatment periods, whereas this consistency was not observed with placebo. Remote data collection proved to be reliable, with a high proportion of collections and measurements performed successfully.

We used UACR change as a surrogate outcome, as early treatment effects are associated with long-term kidney outcomes.^10,17,18,19^ Therefore, UACR is considered to be a reasonably likely surrogate end point for kidney failure and can be used to monitor drug responses.^20^ Prior studies in patients with type 2 diabetes and CKD demonstrated substantial variation in UACR changes with SGLT2 inhibitors, but this variation was also observed in placebo groups.^6,17^ Without re-exposure, it remained unclear whether this reflected random day-to-day variability or a genuine treatment response. In our study, UACR changes during dapagliflozin treatment correlated significantly between periods, suggesting genuine therapeutic heterogeneity. No significant correlation was observed during placebo periods, indicating that UACR variation with placebo administration was random. The imperfect correlation during dapagliflozin treatment suggests other sources of variability, such as day-to-day fluctuations and measurement error.^21^ Using a single urine sample rather than the geometric mean of 3 eliminated the correlation, likely due to random error and reduced precision. This finding underscores the importance of multiple consecutive samples to capture reliable treatment effects and minimize random variability.

Remote data collection was effective, with high success rates for urine samples, capillary blood samples, blood pressure, and body weight measurements. Beyond evaluating SGLT2 inhibitors, this approach holds promise for assessing other medications alone or in combination while reducing hospital visits and maintaining effective data collection and monitoring. Successful implementation in other settings will require compatible devices, a secure electronic data capture environment, and dedicated participant support. The structured use of digital tools, such as those used in our study, streamlined data collection and minimized participant burden, demonstrating its potential for adoption in other settings. Our questionnaire offered initial insights into participants’ experiences with remote data collection but did not address trial duration, data collection frequency, and preferences for at-home vs in-clinic methods. Future trials should use validated questionnaires addressing digital literacy, educational levels, and participant preferences to refine decentralized trial designs and enhance feasibility and engagement.

As the landscape of CKD therapeutics evolves, various novel interventions have emerged and are anticipated to emerge, allowing clinicians to tailor optimal individual or combination therapies. Understanding which patient characteristics are associated with a good or poor UACR response to dapagliflozin is clinically relevant and may help guide treatment decisions. Alternatively, these decisions can be informed by the patient’s response to the treatment. In cases of insufficient response, add-on therapies may be considered. Remote data collection, as used in this study, could facilitate monitoring treatment responses without requiring hospital visits, offering a more patient-friendly experience. This approach enables more frequent monitoring of risk markers, enhancing the precision, effectiveness, and safety of CKD management. It is important to note that SGLT2 inhibitors provide significant benefits across various clinical end points, including reductions in heart failure events, hospitalizations, anemia, gout, hypertension, and mortality,^5,22,23,24,25^ and a lack of UACR response does not necessarily justify discontinuing dapagliflozin treatment. A comprehensive assessment of the patient’s overall risk profile and response across multiple risk markers is essential to determine the ultimate benefit and harms.

### Limitations

This study has limitations. First, the small sample size may limit generalizability but was sufficient for exploring individual treatment responses. Second, the participant population lacked diversity, being predominantly male and all White, reflecting the outpatient clinic’s demographics. This limits the generalizability of the findings despite no intentional bias. Future studies could improve diversity with targeted recruitment strategies. Third, the short duration of dapagliflozin exposure, while sufficient to detect early effects,^8^ leaves the possibility that UACR effects may be larger with longer exposure. With such limited exposure time, the findings remain exploratory and cannot be used to inform personalized treatment decisions regarding progression to hard kidney outcomes. Fourth, remote data collection, although successful, may be less feasible for individuals with limited technical skills or in regions with an unreliable internet infrastructure. While digital literacy was not specifically assessed in our study, participants with various educational backgrounds showed comparable success, suggesting that proper instructions and support can make this approach broadly feasible. Fifth, the Dutch postal system’s efficiency in delivering biomaterials may not be replicable in other countries, potentially affecting data reliability.

## Conclusions

This placebo-controlled, double-blind crossover trial using an n-of-1 approach provided insights into the individual responses of patients with type 2 diabetes to treatment with dapagliflozin. Dapagliflozin reduced UACR compared with placebo, although considerable variability was observed among individuals. These variations, which were consistent after re-exposure, underscore the need for personalized approaches to diabetes and CKD management. Moreover, our research demonstrated the potential advantages of remote data collection, hinting at innovative possibilities in clinical research and patient care. Future studies exploring the implementation of treatment adjustments based on remotely collected data are warranted, as they could inform decentralized optimization of individual treatment regimens, ultimately enhancing the quality of care for patients with type 2 diabetes.

## Notes

Supplement 1.
Trial Protocol and Statistical Analysis Plan

Supplement 2. **eFigure 1.** Changes in systolic blood pressure, body weight, and eGFR during treatment with dapagliflozin and placebo **eFigure 2.** Medication adherence per active treatment day **eMethods.** Questionnaire

Supplement 3.
Data Sharing Statement
