# Supplementary material for: Albuminuria Responses to Dapagliflozin in Patients With Type 2 Diabetes: A Crossover Trial
Source: JAMA Netw Open. 2025 Mar 24;8(3):e251689. doi: 10.1001/jamanetworkopen.2025.1689 (PMC11934004; doi:10.1001/jamanetworkopen.2025.1689)
Supplement: Supplement 1. — Trial Protocol and Statistical Analysis Plan [file jamanetwopen-e251689-s001.pdf]

@Home study

Individual albuminuria lowering response to dapagliflozin in a decentralized clinical trial in patients with type 2 diabetes mellitus and elevated albuminuria

PROTOCOL TITLE: @Home study

**Protocol ID**	2020/1002
**Short title**	**@Home study**
**Version**	1.0
**Date**	April 13, 2021
**Coordinating investigator/ project leader**	Prof. Dr. H.J. Lambers Heerspink<br>Department of Clinical Pharmacology<br>University Medical Center Groningen (UMCG)<br>Hanzeplein 1<br>9700 RB Groningen<br>Tel: 050-3617859<br>E-mail: h.j.lambers.heerspink@umcg.nl
**Principal investigator(s) (in Dutch: hoofdonderzoeker/uitvoerder)**	Prof. Dr. G.D. Laverman<br>Department of Internal Medicine / Nephrology<br>Ziekenhuisgroep Twente<br>Zilvermeeuw 1<br>7609 PP Almelo<br>Tel: 088-7084351<br>E-mail: g.laverman@zgt.nl
**Sponsor (in Dutch: verrichter/opdrachtgever)**	University Medical Center Groningen
**Subsiding parties**	AstraZeneca
**Independent expert(s)**	Dr. B.W. Schot<br>Department of Internal Medicine<br>Ziekenhuisgroep Twente<br>Zilvermeeuw 1<br>7609 PP Almelo<br>Tel: 088-7087076<br>E-mail: b.schot@zgt.nl
**Laboratory sites**	Medlon BV<br>Dr. C.J.A. Doelman<br>Medisch Spectrum Twente / Ziekenhuisgroep Twente<br>7500 KA Enschede<br>Tel: 088-4633566
**Pharmacy**	Hospital Pharmacy<br>Ziekenhuisgroep Twente<br>Zilvermeeuw 1<br>7609 PP Almelo<br>Tel: 088-7085666

TABLE OF CONTENTS

1. INTRODUCTION AND RATIONALE 12

2. OBJECTIVES 15

3. STUDY DESIGN 16

4. STUDY POPULATION 21

4.1. Population (base) 21

4.2. Inclusion criteria 21

4.3. Exclusion criteria 21

4.4. Sample size calculation 23

5. TREATMENT OF SUBJECTS 23

5.1. Investigational product/treatment 23

5.2. Use of co-intervention 24

6. INVESTIGATIONAL PRODUCT 25

6.1. Name and description of investigational product(s) 25

6.2. Summary of finding from non-clinical studies 25

6.3. Summary of findings from clinical studies 25

6.4. Summary of known and potential risks and benefits 27

6.5. Description and justification of route of administration and dosage 28

6.6. Dosages, dosage modifications and method of administration 28

6.7. Preparation and labelling of Investigational Medicinal Product 28

6.8. Drug accountability 29

7. NON-INVESTIGATIONAL PRODUCT 30

7.1. Name and description of non-investigational product(s) 30

7.2. Summary of findings from non-clinical studies 30

7.3. Summary of findings from clinical studies 30

7.4. Summary of known and potential risks and benefits 30

7.5. Description and justification of route of administration and dosage 30

7.6. Dosages, dosage modifications and method of administration 30

7.7. Preparation and labelling of non-investigational medicinal product 30

7.8. Drug accountability 30

8. METHODS 31

8.1. Study parameters/endpoints 31

8.1.1. Main study parameter/endpoint 31

8.1.2. Secondary study parameters/endpoints 31

8.1.3. Other study parameters 31

8.2. Randomization, blinding and treatment allocation 31

8.3. Study procedures 31

8.4. Withdrawal of individual subjects 35

8.5. Replacement of individual subjects after withdrawal 35
8.6. Follow-up subjects withdrawn from treatment 35
8.7. Premature termination of the study 36

9. SAFETY REPORTING 37
9.1. Temporary halt for reasons of subject safety 37
9.2. AEs, SAEs and SUSARs 37
9.2.1. Adverse events (AEs) 37
9.2.2. Serious adverse events (SAEs) 37
9.2.3. Suspected unexpected serious adverse reactions (SUSARs) 39
9.3. Annual safety report 40
9.4. Follow-up of adverse events 41
9.5. [Data Safety Monitoring Board (DSMB) / Safety Committee] 41

10. STATISTICAL ANALYSIS 42
10.1. Primary study parameter(s) 42
10.2. Secondary study parameter(s) 43
10.3. Other study parameters 43
10.4. Interim analysis 43

11. ETHICAL CONSIDERATIONS 44
11.1. Regulation statement 44
11.2. Recruitment and consent 44
11.3. Objection by minors or incapacitated subjects 44
11.4. Benefits and risk assessment, group relatedness 44
11.5. Compensation for injury 45
11.6. Incentives 46

12. ADMINISTRATIVE ASPECTS, MONITORING AND PUBLICATION 47
12.1. Handling and storage of data and documents 47
12.2. Monitoring and Quality Assurance 47
12.3. Amendments 47
12.4. Annual progress report 47
12.5. Temporary halt and (prematurely) end of study report 48
12.6. Public disclosure and publication policy 48

13. STRUCTURED RISK ANALYSIS 49
13.1. Potential issues of concern 49
13.2. Synthesis 52

14. REFERENCES 53

LIST OF ABBREVIATIONS AND RELEVANT DEFINITIONS

ABR	General Assessment and Registration form (ABR form), the application form that is required for submission to the accredited Ethics Committee; in Dutch: Algemeen Beoordelings- en Registratieformulier (ABR-formulier)
AE	Adverse Event
AR	Adverse Reaction
AZ	AstraZeneca
NT-proBNP	N-Terminal Prohormone of Brain Natriuretic Peptide
CA	Competent Authority
CCMO	Central Committee on Research Involving Human Subjects; in Dutch: Centrale Commissie Mensgebonden onderzoek
CRP	C-Reactive Protein
CV	Curriculum Vitae
DSMB	Data Safety Monitoring Board
eGFR	Estimated glomerular filtration rate
EU	European union
EudraCT	European drug regulatory affairs Clinical Trials
GCP	Good Clinical Practice
GDPR	General Data Protection Regulation; in Dutch: Algemene Verordering Gegevensbescherming (AVG)
GLP-1	Glucagon-like Peptide-1
IB	Investigator's Brochure
IC	Informed Consent
IMP	Investigational Medicinal Product
IMPD	Investigational Medicinal Product Dossier

**METC**	**Medical research ethics committee (MREC); in Dutch: medisch-ethische toetsingscommissie (METC)**
**NSAIDs**	**Nonsteroidal anti-inflammatory drugs**
**(S)AE**	**(Serious) Adverse Event**
**SGLT2**	**Sodium Glucose Transport Inhibitor 2**
**SPC**	**Summary of Product Characteristics; in Dutch: officiële productinformatie IB1-tekst**
**Sponsor**	**The sponsor is the party that commissions the organization or performance of the research, for example a pharmaceutical company, academic hospital, scientific organization or investigator. A party that provides funding for a study but does not commission it is not regarded as the sponsor but referred to as a subsiding party.**
**SUSAR**	**Suspected Unexpected Serious Adverse Reaction**
**UAVG**	**Dutch Act on Implementation of the General Data Protection Regulation; in Dutch: Uitvoeringswet AVG**
**UACR**	**Urine albumin-to-creatinine ratio**
**WMO**	**Medical Research Involving Human Subjects Act; in Dutch: Wet Medisch-wetenschappelijk Onderzoek met mensen**

SUMMARY

Rationale:

Persistent increased albuminuria is a strong risk marker for progressive kidney disease and cardiovascular disease in patients with or without diabetes. The degree of albuminuria reduction in the first months of treatment with pharmacological or dietary intervention correlates with the degree of long-term (3 to 4 years) renal or cardiovascular protection. Despite the various available treatments to decrease urinary albumin excretion, residual albuminuria persists in many patients. The high residual albuminuria in a proportion of patients is at least in part explained by suboptimal response to the current treatments (i.e., ACE inhibitor or Angiotensin Receptor Blockers).

Dapagliflozin is a sodium-glucose transport inhibitor and inhibits the reabsorption of glucose in the proximal tubule. This leads to a decrease in fasting plasma glucose and HbA1c in patients with type 2 diabetes. In addition, dapagliflozin administration causes a decrease in blood pressure and body weight and an increase in hematocrit suggestive of a diuretic effect. Previous studies have also demonstrated the albuminuria lowering effects of dapagliflozin in patients with type 2 diabetes mellitus.

Although dapagliflozin markedly slows progression of kidney function decline (and reduces cardiovascular outcomes) on a population level, randomized parallel group trials have suggested a marked variation in the response to dapagliflozin between individual patients. By design, randomized parallel group placebo-controlled clinical trials test the efficacy of new interventions on a population level but do not assess the efficacy of a drug for the individual. Although there is variation in response between patients, parallel group trial does not allow conclusions whether this variation is a true variation in drug response, or measurement or temporal random variation. We therefore propose a cross-over trial with repeated administration (i.e., a series of N=1 trials) to ascertain the individual drug response. This design specifically allows for assessment of drug efficacy and safety at an individual level.

Objective:

- Primary:
  - To determine the individual response to the SGTL2 inhibitor dapagliflozin in urine albumin-to-creatinine ratio (UACR)
- Secondary:
  - To determine the individual response to the SGLT2 inhibitor dapagliflozin

in:

- Systolic blood pressure
- Body weight
- eGFR
- Fasting plasma glucose

Study design:

Randomized placebo-controlled double-blind cross-over N=1 trial. Eligible participants will be invited for screening. After a screening visit, eligible patients will be randomly assigned to a cross-over study consisting of two periods of 1-week treatment with dapagliflozin and two periods of 1-week treatment with placebo in random order with a 1-week wash-out period between every treatment period to avoid cross-over effects. Based on a prior study where patients were exposed to dapagliflozin 10 mg, effects of dapagliflozin on UACR, blood pressure, body weight, eGFR and plasma glucose were fully present after 1 week and returned to baseline 4 days after drug discontinuation. Hence, a 1-week treatment followed by 1 week wash-out is considered sufficient to detect treatment effects.

Study population:

Adult male and female patients with UACR >20 mg/g (2.26 mg/mmol) with type 2 diabetes mellitus treated in primary or secondary healthcare. Subjects will be recruited via general practitioner practices and via the outpatient clinic of the Department of Internal Medicine of the Ziekenhuisgroep Twente, Almelo.

Intervention:

Dapagliflozin 10 mg/day

Main study parameters/endpoints:

Difference in change in first morning void UACR from start to end of treatment between dapagliflozin and placebo exposure.

Nature and extent of the burden and risks associated with participation, benefit and group relatedness:

The efficacy and safety of dapagliflozin is established in multiple parallel randomized controlled trials involving more than 25,000 patients with type 2 diabetes. Urinary tract infections and genital infections are the most frequently reported side effects. Dapagliflozin reduces body weight unlike sulfonylurea derivatives and insulin.

Participants visit the outpatient clinic at three occasions (i.e., a screening visit, a second visit and end of study visit) and have to record body weight and blood pressure at home and collect blood and urine at home.

Blood pressure and body weight are measured at home by the participants using ambulant devices (Withings BPM Connect and Withings Body+, respectively). Participants measure their blood pressure and body weight once daily on 28 and 40 days in total, respectively. Capillary blood will be sampled at home by participants using a BD Microtainer® Contact-Activated Lancet (once daily on 22 days in total). Blood is collected with the Hem-Col® device, which is designed to collect capillary blood drawn with a finger prick. In order to make patients comfortable with the blood collection procedures, they first collect a capillary blood sample at the study site during the second visit under supervision of trained lab technicians. A venous blood sample will also be taken during the second visit in order to compare the clinical chemistry assessments in capillary blood with those measured in venous blood samples (NL70447.100.19). Participants will be asked to draw blood samples at home by a finger prick and send the samples to the laboratory. Participants will collect first morning void urine samples through the PeeSpot® device (once daily on 40 days in total) which allows for decentralized urine collection in a small tube. The urine tubes and blood samples will be sent by regular mail to the laboratory. No other invasive measurements will be executed.

The advantage of an N=1 study is that efficacy of the intervention is vetted for the actual participant. Dapagliflozin is currently marketed in the Netherlands and recommended in patients with type 2 diabetes mellitus and eGFR>45 mL/min/1.73m^2. Patients who show a satisfactory response to dapagliflozin and whose characteristics fulfill the criteria according to which dapagliflozin can be prescribed in clinical practice are offered to receive dapagliflozin after the study. It is expected that the indication for dapagliflozin will be broadened to patients with eGFR 25-45 mL/min/1.73m^2 in the near future. If this occurs, these patients can also be treated on-label in practice.

The expected time investment for participants is 20 hours, including measurements at home. Participants receive restitution of travel costs to visit the outpatient clinic for the screening, randomization and end of study visit. Participants receive no priority in treatment of other diseases in the clinic during this study. Participation in this study is on a free-will base. Participants can keep the body weight scale and blood pressure device at the end of the study.

1. INTRODUCTION AND RATIONALE

The sodium-glucose co-transporter 2 (SGLT2) is found primarily in the S1 segment of the proximal tubule of the kidney and accounts for 90% of the glucose reabsorption in the kidney in order to maintain appropriate glucose levels.[1] SGLT2 transports one ion of Na^+ per molecule of glucose. Because Na^+ is actively extruded by Na/K-ATPase, the extracellular Na^+ concentration substantially exceeds the intracellular concentration. Movement of Na^+ down its electrochemical gradient provides the energy required for active transport of glucose. SGLT2 inhibitors reversely inhibit the SGLT2-transporter, leading to an increase in urinary glucose and sodium excretion by reducing glucose reabsorption in the renal proximal tubule.[2] Initial relatively short-term studies (up to 52 weeks) have shown that SGLT2 inhibitors exert beneficial effects on multiple cardiovascular risk markers including HbA1c, blood pressure, body weight, and uric acid.[2,3] Large clinical trials have shown that SGLT2 inhibitors reduce the risks of heart failure and kidney failure in patients with (or without) type 2 diabetes mellitus (T2DM).[4]

Persistent increased albuminuria is an established risk marker for development of chronic kidney disease and cardiovascular disease events in patients with or without type 2 diabetes.[5] Prior studies have shown that SGLT2 inhibitors reduce the urine albumin-to-creatinine ratio (UACR). These UACR-lowering effects of SGLT2 inhibitors tend to be greater with higher levels of baseline UACR.[2] Prior studies have shown that the SGLT2 inhibitor dapagliflozin reduces UACR in patients with T2DM. The UACR reduction achieved with dapagliflozin was independent of concomitant changes in HbA1c, blood pressure, eGFR or uric acid, suggesting a direct mechanism of action.[2,6,7]

More recently, the DAPA-CKD trial reported the efficacy and safety of dapagliflozin in 4,304 patients with chronic kidney disease with or without type 2 diabetes. The trial demonstrated that dapagliflozin significantly reduced the risk of kidney and cardiovascular endpoints, prolonged survival and reduced the UACR. Furthermore, dapagliflozin was well tolerated in keeping with the established safety profile. No major hypoglycemic events were reported in these patients.[8]

Despite the efficacy of SGLT2 inhibitors to lower cardiovascular risk markers and slow the progression of kidney function decline and prevent cardiovascular events on a population level, there are still many patients who experience clinically relevant outcomes such as dialysis, kidney transplantation or myocardial infarctions while receiving

dapagliflozin or other SGLT2 inhibitors. For example, in the abovementioned DAPA-CKD trial, 197 patients receiving the SGLT2 inhibitor dapagliflozin experienced the primary composite kidney outcome during 2.4 years follow-up, indicating that these patients were sub-optimally protected. This suboptimal protection is likely caused (at least in part) by sub-optimal response to dapagliflozin. Indeed, analyses from parallel-group trials with dapagliflozin have reported a large variation in UACR (or blood pressure or body weight changes) between individual patients in response to dapagliflozin, suggesting that the degree of benefit and response to dapagliflozin substantially varies among individual patients.

However, it should be noted that randomized parallel group placebo controlled clinical trials are used to test the efficacy and safety of new interventions on a population level but do not assess the efficacy of a drug for the individual. Thus, although analyses from parallel group trials have shown variation between patients in response in UACR or blood pressure to dapagliflozin, it cannot be excluded that this variation is due to measurement or temporal random variation. Alternative trial designs are needed and better suited to evaluate the individual response to dapagliflozin. Various clinical trial designs have been developed that better account for the variability between patients than the traditional randomized parallel group double blind controlled trial. The N=1 clinical trial design specifically allows for assessment of drug efficacy and safety at an individual level. In a N=1 trial, the same patient is exposed multiple times in random order to an active compound or placebo intervention in order to directly estimate the effect of the intervention for each individual. The advantage of a N=1 trial is that the efficacy of the intervention is vetted for the actual patient, thereby increasing patient participation and engagement and making the results of the study directly applicable to the participant. Thus, categorizing patients as responders or non-responders by comparing their values with those at baseline is arbitrary and inefficient since the observed change does not guarantee that the patient is responding to the drug. A N=1 trial with the same patient exposed twice to active medication and placebo is the appropriate design to test the individual drug response.

We therefore propose a N=1 trial determining the UACR response to dapagliflozin for each individual specifically. We furthermore hypothesize that the change in UACR in response to dapagliflozin is reproducible upon re-exposure and reflects a true pharmacological response rather than random variation whereas during placebo treatment changes in UACR are thought and do not correlate. A retrospective analysis

from a cross-over study in non-diabetic kidney disease where patients were exposed twice to an ACE inhibitor or Angiotensin Receptor Blocker supports this notion as in that study a strong correlation between the response during the first and second exposure was observed (correlation coefficient between 0.7 and 0.9).[9]

2. OBJECTIVES

- Primary:
  - To determine the individual response to the SGTL2 inhibitor dapagliflozin in urine albumin-to-creatinine ratio (UACR)
- Secondary:
  - To determine the individual response to the SGLT2 inhibitor dapagliflozin in:
    - Systolic blood pressure
    - Body weight
    - eGFR
    - Fasting plasma glucose

3. STUDY DESIGN

A randomized placebo-controlled double-blind cross-over N=1 trial in subjects with type 2 diabetes with albuminuria >20 mg/g (2.26 mg/mmol).

Study execution

The trial will be performed in the remote setting. In a conventional clinical trial, patients have to visit the hospital/clinic multiple times for health check-ups and blood and / or urine collections. This may complicate the feasibility of the trial since not all patients are willing to frequently visit the clinic, which hampers recruitment. In addition, patients may drop out during the trial since the time investment and burden of study visits is higher than originally expected. With the introduction of digital technologies and wearable devices, it becomes possible to assess patients remotely – while they are at home. If used in a clinical trial, it could significantly reduce the number of study visits, time investment for patients, and increase the operational processes of clinical trial conduct.

Some healthcare providers and IT suppliers already offer patients the opportunity to create and manage a personal health record at a central privacy secured server. More and more patients are also keeping their own records, but the scope for combining and sharing all these records is still somewhat limited. Based on a strong believe that people play a vital role in their own health, various parties in the healthcare sector have taken the initiative to set up special platforms to offer people more control over their own health. In this study, we will use such an online platform (Selfcare). Selfcare creates a personal health environment (PHE), a digital tool in which patients can store all their health data in a safe, privacy secured, and independent manner. Up to now, health data has not always been easily accessible, but this PHE allows patients to safely compile and share their own health information online with physicians and researchers.

To take advantage of these developments we will perform this study using digital technologies and ask patients to collect study data at home.

Study design

Subjects with UACR >20 mg/g (2.26 mg/mmol) with a diagnosis of type 2 diabetes who meet the inclusion criteria will be invited for screening (day -14). After the screening visit, eligible patients will proceed to a second visit (day -7). Patients will be randomly assigned to a cross-over study consisting of two periods of 1-week treatment with dapagliflozin and two periods of 1-week treatment with placebo in random order with a 1-week wash-out

period between every treatment period to avoid cross-over effects. Based on a prior study, effects of dapagliflozin on UACR, blood pressure, body weight, fasting plasma glucose and eGFR were fully present after 4 days. We will therefore ask patients to collect urine after 3, 5, 6, and 7 days. Due to day-to-day variation in UACR we are collecting multiple urine samples to increase precision and therefore collect urine at 3, 5, 6, and 7 days. We will also collect urine after 1 day to further delineate the time-course of the response to dapagliflozin and assess if the treatment effect on UACR is directly present after 1 day. Blood pressure and body weight will be recorded and blood samples will be taken at the time points described below and in the table. Overall, a 1-week treatment period is considered sufficient to detect effects on primary and secondary outcomes.

Since data collection will be performed at home using wearable devices and digital technologies, instructions will be provided during the second visit to perform data collection and study procedures at home. Data collection and measurements will be performed in each treatment period as indicated in Figure 1. Bottles with smart caps that allow real-time monitoring of adherence, blood pressure monitors, body analysis scales and study medication will be provided at the second visit. At the second visit, patients receive four numbered bottles with seven tablets in each bottle, according to their randomized treatment schema. Patients will be instructed to take the study medication once daily, in the morning.

Measurements:

- First morning void urine collections for measurement of UACR, sodium, potassium, urea, and osmolality will be performed at:
  - Day -7, -3, -2 and -1
  - Day 1, 3, 5, 6 and 7 of the active periods
  - Day 3, 5, 6 and 7 of the wash-out periods
- Ambulatory blood pressure will be measured by the patients themselves at home at:
  - Day -7, -3, -2 and -1
  - Day 1, 3, 6 and 7 of the active periods
  - Day 6 and 7 of the wash-out periods
- Ambulatory body weight will be measured by the patients themselves at home at:
  - Day -7, -3, -2 and -1
  - Day 1, 3, 5, 6 and 7 of the active periods

  - o Day 3, 5, 6 and 7 of the wash-out periods
- Capillary blood samples for measurement of creatinine, CRP, lipid profile, HbA1c, uric acid, glucose and NT-proBNP will be taken at:
  - o Day -7 (under supervision during the second visit) and -1
  - o Day 1, 6 and 7 of the active periods
  - o Day 6 and 7 of the wash-out periods

  A venous blood sample will also be taken during the second visit in order to compare the clinical chemistry assessments in capillary blood with those measured in venous blood samples.
- Medication will be provided in bottles with smart caps that allow real-time monitoring of adherence.
- Dapagliflozin will be measured by a validated liquid chromatography-mass spectrometry (LC-MS/MS) analysis at day 7 of every active period in capillary blood samples to assess treatment adherence.
- Telephone calls will be made at day 3 and 6 of the first two treatment periods to assess compliance with study procedures.

Questionnaires consisting of 6 multiple choice questions and 2 open questions will be used to assess patient experience and satisfaction to guide and inform future trials and use in clinical practice.

Figure 1: Study design

	Screening visit	Run-in period				Period 1					Wash-out				Period 2/3/4					Wash-out			
Days	-14	-7 (2nd visit)	-3	-2	-1	1	3	5	6	7	3	5	6	7	1	3	5	6	7	3	5	6	7
Urine samples* (PeeSpot)		x	x	x	x	x	x	x	x	x	x	x	x	x	x	x	x	x	x	x	x	x	x
Blood Pressure		x	x	x	x	x	x		x	x			x	x	x	x		x	x			x	x
Body weight		x	x	x	x	x	x	x	x	x	x	x	x	x	x	x	x	x	x	x	x	x	x
Blood samples** (Hem-Col)		x			x	x			x	x			x	x	x			x	x			x	x
Glucose (Strips)		x			x	x			x	x			x	x	x			x	x			x	x
Treatment adherence						x	x	x	x	x	x	x	x	x	x	x	x	x	x	x	x	x	x

(MEMS)																							
Dapagliflozin concentration (LC-MS/MS)										x									x				
Questionnaire																							x

* UACR, sodium, potassium, urea, and osmolality will be measured

**Creatinine, CRP, lipid profile, HbA1c, uric acid, and NT-proBNP will be measured

Patients connect their blood pressure monitor and body analysis scale to the corresponding mobile application, which in turn is linked to Selfcare platform. In this way, data will be automatically transferred to Selfcare platform. Patients can also register adverse events in Selfcare. Selfcare creates a personal health environment (PHE), a digital tool in which patients can store all their health data in a safe and independent manner. These data are integrated on a single independent platform. This unique dynamic dashboard forms a reliable basis on which health data can be monitored. At the end of the study, blood pressure and body weight data will be downloaded from Selfcare and along with the biochemical data measured in the central laboratory incorporated in the database.

Selfcare provides the best possible data protection. Selfcare meets the Dutch standard for information security in the healthcare sector (NEN 7510), which relates to the formulation, registration and monitoring of information security. This standard entails: ‘Ensuring the availability, integrity and confidentiality of all information needed to provide patients with responsible care.’

Selfcare users have full ownership of their personal health data at all times. Only if the user gives explicit consent, third parties can be granted access to the data. Communication with peers in the in the community and data exchange (if permitted) takes place in a secure environment. Selfcare guarantees privacy whether a patient uses a computer, tablet or smartphone.

When users open Selfcare dashboard from a new device, they are asked to provide a second piece of information (in addition to their username and password) to prove their identity. This is an additional step taken by Selfcare to prevent abuse and provide personal safety.

Selfcare uses HTTPS, a protocol that is used for the secure handling of request between a client (browser) and server (web server). Data are encrypted, making it

virtually impossible for someone without proper authorization to access the data. The selfcare servers are hosted at a national level by independent foundations to ensure privacy.

The Selfcare data are subjected to independent supervision by leading authorities in the field of privacy and patient protection. All personal user data are stored on a server, which is located in the country of registration. Ownership of this server lies with a legal entity that is separated from the commercial organization Selfcare. This means that the data are never accessible to third parties and can never be sold to third parties. This ensures that only the end user determines what happens to his or her data.

4. STUDY POPULATION

4.1. Population (base)

The study population will consist of adult male and female patients with UACR >20 mg/g (2.26 mg/mmol) with T2DM treated in primary or secondary healthcare. Subjects will be recruited via general practitioner practices or via the outpatient clinic of the Department of Internal Medicine of the Ziekenhuisgroep Twente, Almelo.

4.2. Inclusion criteria

In order to be eligible to participate in this study, a subject must meet all of the following criteria:

- Age ≥18 years
- Diagnosis of type 2 diabetes mellitus
- Urinary albumin-to-creatinine ratio >20 mg/g (2.26 mg/mmol)
- eGFR >30 ml/min/1.73m^2
- Willing to sign informed consent

4.3. Exclusion criteria

A potential subject who meets any of the following criteria will be excluded from participation in this study:

- Diagnosis of type 1 diabetes
- Prior treatment with SGLT2 inhibitor in the four weeks prior to randomization
- History of severe hypersensitivity or contraindications to dapagliflozin
- Unable to monitor blood pressure / body weight or handle digital technologies
- History of non-adherence to medical regimens or unwillingness to comply with the study protocol
- Participation in any clinical investigation within 3 months prior to initial dosing
- Unstable or rapidly progressing renal disease
- Severe hepatic impairment (Child-Pugh class C) as determined by the treating physician.
- Active malignancy

- Any medication, surgical or medical condition which might significantly alter the absorption, distribution, metabolism, or excretion of medications including, but not limited to any of the following:
    - History of active inflammatory bowel disease, within the last six months.
    - Major gastrointestinal tract surgery as decided by the treating physician
    - Pancreatitis within the last six months.
    - Evidence of serious hepatic disease as determined by the treating physician
    - Evidence of urinary obstruction or difficulty in voiding at screening.
- Confirmed lactose intolerance demonstrated with a lactose intolerance test.
- Donation or loss of 400 mL of blood within 8 weeks prior to initial dosing
- History of drug or alcohol abuse within the 12 months prior to dosing, or evidence of such abuse as indicated by the laboratory assays conducted during the screening
- Any surgical or medical condition, which in the opinion of the investigator, may place the patient at higher risk from his/her participation in the study, or is likely to prevent the patient from complying with the requirements of the study or completing the study.
- Current pregnancy or breast feeding / attempting to conceive.
- Women of childbearing potential (WOCBP):
    - WOCBP who are unwilling or unable to use an acceptable method of contraception to avoid pregnancy throughout the study and for up to 4 weeks after the last dose of study drug in such a manner the risk of pregnancy is minimized.
    - WOCBP must have a negative serum or urine pregnancy test result (minimum sensitivity 25 IU/L or equivalent of HCG) within 0 to 72 hours before the first dose of study drug.

WOCBP comprises women who have experienced menarche and who have not undergone successful surgical sterilization (hysterectomy, bilateral tubal ligation, or bilateral oophorectomy) or who are not post-menopausal (see definition below). The following women are WOCBP:

- Women using the following methods to prevent pregnancy: oral contraceptives, other hormonal contraceptives (vaginal products, skin patches, or implanted or injectable products), or mechanical products such as intrauterine devices or barrier methods (diaphragm, condoms, spermicides).
- Women who are practicing abstinence.
- Women who have a partner who is sterile (e.g. due to vasectomy).

Post-menopause is defined as:

- Women who have had amenorrhea for $\geq$12 consecutive months (without another cause) and who have a documented serum follicle-stimulating hormone (FSH) level >35 mIU/mL.
- Women who have irregular menstrual periods and a documented serum FSH level >35 mIU/mL.
- Women who are taking hormone replacement therapy (HRT).

4.4. Sample size calculation

The sample size for this study is based on the hypothesis that the response to dapagliflozin is reproducible upon re-exposure and thus correlates between the first and second exposure while no correlation will be detected during placebo patients. A prior retrospective study found that during intervention in the renin-angiotensin-system responses in proteinuria correlate with correlation coefficients ranging between 0.7 to 0.9.[9] Since this was a retrospective study, we assume a more conservative correlation coefficient of 0.6. A sample size of 20 patients provides >80% power (alpha 0.05) to detect a correlation coefficient of 0.6.

5. TREATMENT OF SUBJECTS

5.1. Investigational product/treatment

Dapagliflozin tablets and matching placebos will be purchased and provided by AstraZeneca (AZ). Patients take 10 mg dapagliflozin or matching placebo once

daily in the morning according to a randomized treatment scheme. At the second visit, study medication will be dispensed in standard medicine bottles with a cap that allows real-time monitoring of adherence. Patients receive four numbered bottles with seven tablets in each bottle, according to their randomized treatment schema. Study medication is received at the study site by a designated person, handled and stored safely and properly according to the instructions specified on the drug labels. Study medication is kept in a secured location. Storage conditions are adequately monitored. Subjects are asked to return all unused study drug and packaging at the end of the study or at the time of study drug discontinuation. Unused drugs are destroyed by the pharmacy department at the end of the study.

5.2. Use of co-intervention

Use of following treatments is NOT allowed after the start of the study as these medications may interfere with the evaluation of safety, tolerability and/or efficacy.

- Pioglitazone
- Nonsteroidal anti-inflammatory drugs (NSAIDs)
- Loop or thiazide diuretics, unless stable dose for ≥4 weeks
- Glucagon-like Peptide-1 (GLP-1) Receptor agonists, unless stable dose for ≥4 weeks

Patients who are receiving such medication(s) should be excluded, when dapagliflozin is used in combination with insulin or an insulin secretagogue, such as a sulphonylurea, a reduction of the dose of insulin or insulin secretagogue may be considered to reduce the risk of hypoglycemia.

6. INVESTIGATIONAL PRODUCT

6.1. Name and description of investigational product(s)

Drug name: dapagliflozin (Forxiga, AstraZeneca, EU/1/12/795/009); Chemical structure: (2S,3R,4R,5S,6R)-2-[4-chloro-3-(4-ethoxybenzyl)phenyl]-6-(hydroxymethyl)tetrahydro-2H-pyran-3,4,5-triol;

6.2. Summary of finding from non-clinical studies

This is not applicable as dapagliflozin is already registered for the treatment of cardiovascular disease and type 2 diabetes in humans.

6.3. Summary of findings from clinical studies

Dapagliflozin

SGLT2, located in the proximal tubule of the kidney, is an effective transporter system that is responsible for reabsorption of glucose and sodium. Dapagliflozin is an SGLT2 inhibitor that reversely inhibits the SGLT2 transporter. This leads to enhanced glucose and sodium excretion and reductions in HbA1c, plasma volume, body weight and blood pressure. Previously, we also showed the UACR-lowering effects of dapagliflozin in a prospective randomized controlled trial in patients with type 2 diabetes and ACR >100 mg/g on a stable dose of an ACE inhibitor or angiotensin receptor blocker.[10] A total of 33 patients completed the study. Dapagliflozin 10 mg, as compared to placebo, reduced 24-hour urinary albumin excretion (24h UAE) rate by 36.2% (95% CI 22.9 – 47.2; p <0.001). Furthermore, the eGFR fell by 5.3 (95% CI 2.7 – 8.0). All effects were reversible directly after treatment discontinuation.

In the CREDENCE trial, patients with type 2 diabetes and albuminuric chronic kidney disease were assigned to receive canagliflozin, an oral SGLT2 inhibitor, at a dose of 100 mg daily or placebo.[9] The primary outcome was a composite of end-stage kidney disease (dialysis, transplantation, or a sustained eGFR of <15 mL per minute per 1.73 m^2), a doubling of the serum creatinine level, or death from renal or cardiovascular causes. The trial was stopped early after a planned interim analysis on the recommendation of the data and safety monitoring committee. At that time, 4,401 patients had undergone randomization, with a median follow-up of 2.62 years. The relative risk of the primary outcome was 30% lower in the canagflilozin group than in the placebo group, with event rates of 43.2 and 61.2

per 1000 patient-years, respectively (HR 0.70; 95% CI 0.59 – 0.82; p = 0.00001). The relative risk of the renal-specific composite of end-stage kidney disease, a doubling of the creatinine level, or death from renal causes was lower by 34% (HR 0.66; 95% CI 0.53 – 0.81; p <0.001), and the relative risk of end-stage kidney disease was lower by 32% (HR 0.68; 95% CI 0.54 – 0.86; p = 0.002).

A cardiovascular outcomes study (DECLARE) was conducted to determine the effect of 10 mg dapagliflozin compared with placebo in 17,160 patients with type 2 diabetes mellitus with or without established cardiovascular disease to evaluate the effect on cardiovascular and renal events.[11] 8,582 patients were randomized to dapagliflozin 10 mg and 8,578 to placebo and were followed for a median of 4.2 years. Dapagliflozin reduced the incidence of events of the composite of confirmed sustained eGFR decrease, end-stage renal disease, renal or cardiovascular death. The hazard ratio for time to nephropathy was 0.53 (95% CI 0.43 – 0.66) for dapagliflozin versus placebo. In addition, dapagliflozin reduced the new onset of sustained albuminuria (HR 0.79 [95% CI 0.72 – 0.87]) and let to greater regression of macroalbuminuria (HR 1.82 [95% CI 1.51 – 2.20]) compared with placebo.

The DAPA-CKD trial was conducted in 4,304 patients with CKD, with or without type 2 diabetes.[8] Participants were randomly assigned to receive dapagliflozin (10 mg once daily) or placebo. The trial was recommended to stop by the independent data monitoring committee because of efficacy of dapagliflozin in patients with CKD. The primary outcome was a composite of a sustained decline in the eGFR of at least 50%, end-stage kidney disease, or death from renal or cardiovascular causes. Over a median of 2.4 years, a primary outcome event occurred in 197 of 2152 participants (9.2%) in the dapagliflozin group and 312 of 2152 participants (14.5%) in the placebo group (HR 0.61, 95% CI 0.51 – 0.72; p <0.001). The number needed to treat to prevent one primary outcome event was 19 (95% CI 15 – 27). The hazard ratio for the composite of a sustained decline in the eGFR of at least 50%, end-stage kidney disease, or death from renal causes was 0.56 (95% CI 0.45 – 0.68; p <0.001). No major hypoglycemic events were reported. The effects of dapagliflozin were similar in participants with type 2 diabetes and in those without type 2 diabetes. The known safety profile of dapagliflozin in patients with CKD, regardless of the presence or absence of diabetes, was confirmed.

However, despite the marked effects of SGLT2 inhibitors to reduce the risks of cardiovascular and kidney complications on a population level, not all patients benefit to the same degree. For example, in the DAPA-CKD trial, 197 patients receiving the SGLT2 inhibitor dapagliflzoin experienced the primary composite kidney outcome during 2.4 years follow-up, indicating that these patients were sub-optimally protected.

The current study will therefore further assess, using a dedicated clinical trial design, to study the individual response to dapagliflozin.

6.4. Summary of known and potential risks and benefits

The primary assessment of safety and tolerability of dapagliflozin was conducted in a pre-specified pooled analysis of 13 short-term (up to 24 weeks) placebo-controlled studies with 2,360 subjects treated with dapagliflozin 10 mg and 2,295 with placebo. Of the 17,160 patients in the dapagliflozin cardiovascular outcome study, 8,574 patients received dapagliflozin 10 mg and 8,569 received placebo for a median exposure time of 48 months. In total, there were 30,623 patient-years of exposure to dapagliflozin. The most frequently reported adverse reactions across the clinical studies were genital infections.

The overall incidence of adverse events (short-term treatment) in subjects treated with dapagliflozin 10 mg was similar to placebo. Few adverse events led to discontinuation of treatment and were balanced across study groups. The most commonly reported events leading to discontinuation in patients treated with dapagliflozin 10 mg were increased blood creatinine (0.4%), urinary tract infections (0.3%), nausea (0.2%), dizziness (0.2%), and rash (0.2%).

The most frequently reported adverse reaction was hypoglycemia, which depended on the type of background therapy used in each study. The frequency of minor episodes of hypoglycemia was similar between treatment groups, including placebo, with the exceptions of studies with add-on sulphonylurea and add-on insulin therapies. Combination therapies with sulphonylurea and add-on insulin had higher rates of hypoglycemia.

In the 13-study safety pool, vulvovaginitis, balanitis and related genital infections were reported in 5.5% and 0.6% of subjects who received dapagliflozin 10 mg and placebo, respectively. Most infections were mild to moderate, and subjects responded to an initial course of standard treatment and rarely resulted in discontinuation from dapagliflozin treatment. These infections were more frequent in females (8.4% and 1.2% for dapagliflozin and placebo, respectively), and subjects with a prior history were more likely to have a recurrent infection.

In the dapagliflozin cardiovascular outcomes study, the number of patients with serious adverse events of genital infections were few and balanced: 2 patients in each of the dapagliflozin and placebo groups.

In subjects with severe hepatic impairment (Child-Pugh class C), it was reported that mean C_{max} and AUC of dapagliflozin were 40% and 67% higher than matched healthy controls, respectively. In this study, severe hepatic impairment is an exclusion criterion only when it is known, since the Child-Pugh score is usually not determined in general practice.

6.5. Description and justification of route of administration and dosage

Dapagliflozin is absorbed from the digestive tract and can therefore be orally administered.

6.6. Dosages, dosage modifications and method of administration

Dapagliflozin will be administered in a dose of 10 mg/day.

6.7. Preparation and labelling of Investigational Medicinal Product

AZ supply chain provides the Investigational Medicinal Product Dapagliflozin 10 mg and matching placebo in unlabeled bottles of 35 tablets/bottle. Design of labels, labelling, QP release and distribution of finished packs is the responsibility of the University Medical Center Groningen. Storage will take place at the pharmacy unit of the Ziekenhuisgroep Twente in accordance with relevant GMP guidelines (Good Manufacturing Practice 2003/94/EG).

6.8. Drug accountability

All study medication will be stored at room temperature at the pharmacy department. Bottles with smart caps that allow real-time monitoring of adherence will be provided to eligible patients. At the second visit, patients receive four numbered bottles with seven tablets in each bottle, according to their randomized treatment schema. Non-used medication will be returned to the pharmacy department where it will be destructed.

7. NON-INVESTIGATIONAL PRODUCT

7.1. Name and description of non-investigational product(s)

N/A

7.2. Summary of findings from non-clinical studies

N/A

7.3. Summary of findings from clinical studies

N/A

7.4. Summary of known and potential risks and benefits

N/A

7.5. Description and justification of route of administration and dosage

N/A

7.6. Dosages, dosage modifications and method of administration

N/A

7.7. Preparation and labelling of non-investigational medicinal product

N/A

7.8. Drug accountability

Medication will be provided in standard medicine bottles with the MEMS® (Medication Electronic Monitoring System) Cap (AARDEX Ltd, Union City, CA, USA) which is a customizable medication package which records and stores up to 4,000 dosing events. The cap fits on standard medicine bottles and with integrated microcircuits, the child-resistant MEMS® Cap records the date and time whenever a patient opens a vial. The stored information can be transferred at any time through the MEMS® Reader to the adherence software for immediate analysis and interpretation.

8. METHODS

8.1. Study parameters/endpoints

8.1.1. Main study parameter/endpoint

To determine the individual response to the SGLT2 inhibitor dapagliflozin in urine albumin-to-creatinine ratio (UACR)

8.1.2. Secondary study parameters/endpoints

To determine the individual response to the SGLT2 inhibitor dapagliflozin in:

- Systolic blood pressure
- Body weight
- eGFR
- Fasting plasma glucose

8.1.3. Other study parameters

N/A

8.2. Randomization, blinding and treatment allocation

Treatment sequence of dapagliflozin and placebo will be randomized by the pharmacy unit of the University Medical Center Groningen. A computer-generated randomized code will be used. The pharmacy of the University Medical Center Groningen will store the randomization code.

8.3. Study procedures

- Physical examination

  Patients will be subjected to physical examination by the investigator before inclusion. This physical examination entails a routine investigation of heart, lungs and abdomen.

Data collection will be performed at home using wearable devices and digital technologies. Data collection and measurements will be performed in each treatment period as indicated in Figure 1. The following devices will be used to measure bio-physical/anthropomorphic variables:

- Body weight measurements

Body weight will be measured by the Withings® Body+ (Withings, Issy-les Moulineux, France) device, which is a clinically validated advanced Wi-Fi smart scale that performs highly accurate bioelectrical impedance analysis (weight, body fat & water percentage, plus muscle & bone mass). Patients will be instructed to always weigh themselves under identical conditions. Preferably, weighing will take place in the morning, after showering or bathing, and with dry feet, but before breakfast. Patients will also be instructed to stand up straight and stand still.

- Blood pressure measurements

The Withings® BPM Connect device will be used to monitor blood pressure. This clinically validated Wi-Fi blood pressure monitor provides medically accurate blood pressure and heart rate measurements compliant with European medical device standards. The results are within the margin of acceptance defined by the internationally recognized evaluation standard of blood pressure monitors ANSI/AAMI/ISO 81060-2:2013, EN ISO 81060-2:2014, developed by the European Society of Hypertension, British Hypertension Society and Association for the Advancement of Medical Instrumentation/American Heart Association. Both of the Withings devices will be connected with a smartphone app in order to transfer the data to Selfcare platform.

Prior to starting home blood pressure monitoring, patients will be instructed to measure their blood pressure in both arms to determine which arm should be used for future measurements. The arm that gives the higher systolic reading should be used for all future testing. Patients should place the cuff on their arm with the lower edge of the cuff approximately 2cm above the bend in their elbow. The center of the inflatable bladder should be positioned over the brachial artery on the arm's inferior surface. The forearm should be supported on a firm surface and should be at the level of the lower end of the breastbone. No tight or restrictive clothing should be worn around the arm. Patients should be seated, remain silent and be at rest for a minimum of five minutes before taking a measurement and should not have smoked, eaten, drunk a caffeinated drink or undertaken physical activity within the past thirty minutes. Patients should also avoid measuring their blood pressure with

a full bladder. Measurements should be taken in silence when the patient is relaxed, with both feet flat on the floor and their back and arm supported. Many patients automatically cross their legs, which raises their blood pressure, so it is particularly important to emphasize the need for the patient to uncross their legs when taking their blood pressure.[13] For each BP recording, three consecutive measurements are taken, at least 1 minute apart with the person seated. BP is recorded once daily.[14,15]

Blood and urine will be sampled with dedicated devices:

- <u>Blood sampling:</u>

  Capillary blood will be sampled using a BD Microtainer® Contact-Activated Lancet (Franklin Lakes, New Jersey, USA). Blood is collected with the Hem-Col® device, which is designed to collect capillary blood drawn with a finger prick. Hem-Col is a microtube containing an anticoagulant and a preservation buffer to enhance analyte stability in whole blood. The Hem-Col® device containing 17 USP/mL lithium heparin dissolved in 150 µL preservation fluid (Hem-Col, Labonovum, Limmen, the Netherlands) will be used. Hem-Col tubes have the size of regular blood collection tubes (13 x 75 mm) and are made of polyethylene, with a pierceable cap made of thermoplastic elastomers. All tubes contain a liquid barrier, the inner part serves as a liquid barrier by preventing loss of Hem-Col conservation fluid and the outer part is used as a scoop to collect blood from a finger prick. Hem-Col lithium heparin will be used for analyses of creatinine, C-reactive protein (CRP), lipid profile, HbA1c, uric acid and the N-terminal prohormone of brain natriuretic peptide (NT-proBNP). The Hem-Col device can be sent to the laboratory for analysis by regular post.

  Capillary blood sampled by BD Microtainer® Contact-Activated Lancet will also be used to determine fasting plasma glucose. If a patient already has an eligible glucose meter, this device can be used in order to determine fasting plasma glucose. In this case, patients have to manually enter their blood glucose results in Selfcare platform. If a patient does not already have a glucose meter, an Accu-Chek® Instant System (Roche Diagnostics, GmbH, Mannheim, Germany) device with appropriate test

strips (Accu-Chek® Instant) will be provided during the second visit. The accuracy of the Accu-Chek® Instant System is guaranteed by the makers of the Accu-Chek products, fulfilling the requirements of the ISO 15197:2013/EN ISO 15197:2015. Once connected to Google fit, the meter automatically logs blood glucose results and wirelessly transfers them to Selfcare platform.

- <u>First morning void urine collection</u>

  Urine will be collected with the PeeSpot® (Hessels+Grob, Apeldoorn, the Netherlands) device, which is a validated tool for collection and conservation of small amounts of urine. It consists of a urine absorption pad, a holder, a tube and a lid. Patients can void over the absorption pad while placed in the holder. After voiding, the pad and holder are placed in the tube, closed with the lid and kept in the refrigerator until sending to the laboratory. By adding an inert hygroscopic polymer, the pad easily absorbs 1.2 mL urine and by adding various preservatives, urine is preservable for 4 days. After completing the urine collections, the PeeSpots are placed in a safety bag and sent to the central laboratory in an envelope for biological materials (PolyMed, DaklaPack, Europe). We will use the PeeSpot to determine the first morning void UACR, sodium, potassium, urea, and osmolality. The PeeSpot has been validated for measurement of urinary albumin and creatinine.

- <u>Treatment adherence</u>

  Medication will be provided in standard medicine bottles with the MEMS® (Medication Electronic Monitoring System) Cap (AARDEX Ltd, Union City, CA, USA), which is a customizable medication package which records and stores up to 4,000 dosing events. The cap fits on standard medicine bottles and with integrated microcircuits, the child-resistant MEMS® Cap records the date and time whenever a patient opens a vial. The stored information can be transferred at any time through the MEMS® Reader to the adherence software for immediate analysis and interpretation.

- <u>Laboratory measurements</u>

  Dapagliflozin concentration measurements will be performed at the University Medical Center Groningen using liquid chromatography mass-spectrometry (LC-MS/MS) in capillary blood samples. First morning void

UACR, sodium, potassium, urea, and osmolality measurements will also be performed at the laboratory of the University Medical Center Groningen. Blood (creatinine, CRP, lipid profile, HbA1c, uric acid and NT-proBNP) measurements will be performed at the laboratory (Medlon BV) of the Ziekenhuisgroep Twente. Residual urine and blood plasma samples will be stored at the University Medical Center Groningen in the context of this study to allow for future exploratory biomarker analyses to further study the effects of dapagliflozin on type 2 diabetes, albuminuria and kidney function. Samples will be stored with the same patient number used in the study database. The samples are only accessible to the principal investigator and other delegated members of the study team. No biobank is made.

8.4. Withdrawal of individual subjects

Subjects can leave the study at any time for any reason if they wish to do so without any consequences. The investigator can decide to withdraw a subject from the study for urgent medical reasons.

8.5. Replacement of individual subjects after withdrawal

Patients that meet the inclusion criteria will be invited for participation in the study. When a patient agrees to participate in the study, patients will be invited for screening and informed consent is signed. After the screening visit, eligible patients will proceed to the second visit. When patients decide to withdraw after start of the treatment period, they will be replaced so that twenty patients will complete the full study protocol.

8.6. Follow-up subjects withdrawn from treatment

Subjects who withdraw from the study will be followed up according to the routine terms of patient care at the outpatient clinic or referred back to their general practitioner. Patients who show a satisfactory response to dapagliflozin and whose characteristics fulfill the criteria according to which dapagliflozin can be prescribed in clinical practice are offered to receive dapagliflozin after the study.

8.7. Premature termination of the study

There are no predefined criteria for premature termination of the study. If, however, during the conductance of the study information becomes available showing that continuation of the study would result in a significant safety risk for the patients, the principal investigator and project leader will decide to terminate the study.

9. SAFETY REPORTING

9.1. Temporary halt for reasons of subject safety

In accordance to section 10, subsection 4, of the WMO, the sponsor will suspend the study if there is sufficient ground that continuation of the study will jeopardize subject health or safety. The sponsor will notify the accredited METC without undue delay of a temporary halt including the reason for such an action. The study will be suspended pending a further positive decision by the accredited METC. The investigator will take care that all subjects are kept informed.

9.2. AEs, SAEs and SUSARs

9.2.1. Adverse events (AEs)

Adverse events are defined as any undesirable experience occurring to a subject during the study, whether or not considered related to dapagliflozin. All adverse events reported spontaneously by the subject or observed by the investigator or his staff will be recorded. Reporting from investigator to sponsor will be done by emailing to AE Mailbox Clinical Trial (TCS) (AEMailboxClinicalTrialTCS@astrazeneca.com).

9.2.2. Serious adverse events (SAEs)

A serious adverse event is any untoward medical occurrence or effect that:

- results in death;
- is life threatening (at the time of the event);
- requires hospitalization or prolongation of existing inpatients' hospitalization;
- results in persistent or significant disability or incapacity;
- is a congenital anomaly or birth defect; or
- any important medical event that did not result in any of the outcomes listed above due to medical or surgical intervention but could have been based upon appropriate judgement by the investigator.

An elective hospital admission will not be considered as a serious adverse event.

The investigator will report all SAEs to the sponsor without undue delay after obtaining knowledge of the events. The sponsor will report the SAEs through the web portal *ToetsingOnline* to the accredited METC that approved the protocol, within 7 days of first knowledge for SAEs that result in death or are life threatening followed by a period of maximum of 8 days to complete the initial preliminary report. All other SAEs will be reported within a period of maximum 15 days after the sponsor has first knowledge of the serious adverse events.

During the study, the sponsor will also report all related SAEs to AZ and provides AZ, at latest within two weeks after database lock, a final unblinded line listing of all related SAEs (including SUSARs) notified to regulatory authority and AZ during the study for safety event reconciliation purpose.

During study the sponsor shall:

- Provide AZ Individual Case Safety Reports (ICSRs), initial and follow-up information, as they occur for related SAEs:
  - Unblinded Suspected Unexpected Serious Adverse Reactions (SUSARs)
  - Suspected Serious Adverse Reactions (SSARs)
- Accept to be contacted by AZ regarding any safety reports including contradictory information or missing data. Sponsor is expected to respond to any such queries in a timely manner.
- Provide AZ with any emerging safety issues, unanticipated problems or actions as a result of a safety signal with AZ study drug within 24 hours of knowledge.
- Provide AZ with a copy of their DSUR (Development Safety Update Report) in those cases the safety reference information used by the sponsor, is inconsistent with AZ IB and Local Label. In these rare circumstances, it is provided for informational purposes only. It is made available in parallel of it being distributed externally.

9.2.3. Suspected unexpected serious adverse reactions (SUSARs)

Adverse reactions are all untoward and unintended responses to an investigational product related to any dose administered.

Unexpected adverse reactions are SUSARs if the following three conditions are met:

1. the event must be serious (see chapter 9.2.2);
2. there must be a certain degree of probability that the event is a harmful and an undesirable reaction to the medicinal product under investigation, regardless of the administered dose;
3. the adverse reaction must be unexpected, that is to say, the nature and severity of the adverse reaction are not in agreement with the product information as recorded in:
    - Summary of Product Characteristics (SPC) for an authorized medicinal product;
    - Investigator’s Brochure for an unauthorized medicinal product.

The sponsor will report expedited the following SUSARs through the web portal *ToetsingOnline* to the METC

- SUSARs that have arisen in other clinical trials of the same sponsor and with the same medicinal product, and that could have consequences for the safety of the subjects involved in the clinical trial was assessed by the METC.

The remaining SUSARs are recorded in an overview list (line-listing) that will be submitted once every half year to the METC. This line-listing provides an overview of all SUSARs from the study medicine, accompanied by a brief report highlighting the main points of concern.
The expedited reporting of SUSARs through the web portal Eudravigilance or ToetsingOnline is sufficient as notification to the competent authority.

The sponsor will report expedited all SUSARs to the competent authorities in other Member States, according to the requirements of the Member States.

The expedited reporting will occur not later than 15 days after the sponsor has first knowledge of the adverse reactions. For fatal or life-threatening cases, the term will be maximal 7 days for a preliminary report with another 8 days for completion of the report.

There will be three sets of emergency code breaks, one set will be retained by the project leader, one set will be retained by the principal investigator and a set will be forwarded to the pharmacy. The blinded emergency code break contains the details of drug treatment. In an emergency, the code can be unblinded to identify the treatment given to that subject. Unblinding is not to be performed for any reason, other than an emergency where unblinding is required. When the Investigator removes the scratch-off covering he/she must note the date, time and reason for removing it and record this information in the Comments section of the CRF/ on the code-break card (source data). He/she must also immediately inform the project leader and the principal investigator that the code has been broken. Even though the code is broken, any blood samples for safety or pharmacodynamic assessments will continue to be drawn, for at least 24hr following the last dose as long as doing so will not compromise subject welfare.
It is the responsibility of the Investigator to ensure that there is a procedure in place to allow access to the code break cards in case of emergency. Study drug must be discontinued after unblinding, but the subject will be followed until resolution of the adverse event. At the conclusion of the study, the occurrence of any emergency code breaks will be determined after return of all code break reports.

9.3. Annual safety report

In addition to the expedited reporting of SUSARs, the sponsor will submit, once a year throughout the clinical trial, a safety report to the accredited METC,

competent authority, and competent authorities of the concerned Member States.

This safety report consists of:

a list of all suspected (unexpected or expected) serious adverse reactions, along with an aggregated summary table of all reported serious adverse reactions, ordered by organ system, per study;

a report concerning the safety of the subjects, consisting of a complete safety analysis and an evaluation of the balance between the efficacy and the harmfulness of the medicine under investigation.

9.4. Follow-up of adverse events

All AEs will be followed until they have abated, or until a stable situation has been reached. Depending on the event, follow-up may require additional tests or medical procedures as indicated, and/or referral to the general physician or a medical specialist. SAEs need to be reported till end of study within the Netherlands, as defined in the protocol.

9.5. [Data Safety Monitoring Board (DSMB) / Safety Committee]

A DSMB is not needed for this study, given the extensive experience with dapagliflozin in the enrolled population (safety data from clinical trials available in more than 30,000 patients).

10. STATISTICAL ANALYSIS

10.1. Primary study parameter(s)

The change from baseline in UACR will be calculated using the values collected at day 5, 6 and 7 of each treatment and wash-out period. Because of the skewed distribution UACR will be log-transformed before analysis. The geometric mean of these 3 values (at baseline to end of treatment) will be calculated as $\log(UACR_{end}/UACR_{baseline})$ and the geometric mean change from baseline will be determined.

The study has two cycles for two treatment periods (dapagliflozin and placebo). For calculation of response, the difference in UACR change between dapagliflozin and placebo will be calculated during the first and second cycle. To this end a linear mixed effects model with random intercepts and random slopes for treatment will be used to determine the response during each period. Fixed effects include treatment sequence, and treatment cycle. Baseline log transformed UACR may be added as covariate. Appropriate contrasts will be implemented to assess the placebo substracted response during the first and second cycle and to correlate the placebo adjusted UACR responses to dapagliflozin (fitted on the log-scale) during the two treatment periods. The variance-covariance pattern of the mixed effects model is assumed to be unstructured. If the model does not converge less stringent models will be considered.

To determine if there is within individual variation in treatment response, we will fit a linear mixed model with correlated random intercepts and random slopes for treatment and include treatment period as a fixed effect. Subsequently, we will compare (using a maximum-likelihood ratio fit test) this model against a random intercept only model with treatment and treatment period as fixed effects.

In an additional supportive analysis, we will also dichotomize response. If the placebo adjusted difference in both cycles is greater than 20% in favor of dapagliflozin (using the linear mixed effects model), the patient will meet the response definition. We will then perform a McNemar test for the proportion of concordant response (response during the two cycles or no response during

the two cycles) versus discordant responses (i.e., response first cycle and no response second cycle or vice versa). The proportion of responders will also be calculated if UACR response is defined as a 30% difference in UACR. The 20% and 30% response threshold is based on prior studies of UACR response.

10.2. Secondary study parameter(s)

The secondary outcome parameters will be analyzed in the same way as the primary outcome. For blood pressure, body weight and clinical chemistry laboratory parameters, the average of the readings obtained at day 6 and 7 of the active and wash-out period will be used to calculate the change.

To visualize time courses of the effect in individual patients, all blood pressure and body weight readings collected throughout the study will be used.

10.3. Other study parameters

Treatment adherence will be calculated by determining the proportion of tablets taken each period. In addition, plasma dapagliflozin concentration will be assessed.

10.4. Interim analysis

N/A

11. ETHICAL CONSIDERATIONS

11.1. Regulation statement

The study will be conducted according to the principles of the Declaration of Helsinki (Fortaleza, 2013) and in accordance with the Medical Research Involving Human Subjects Act (WMO). The Medical Ethical Committee of the University Medical Center in Groningen has to approve the study.

11.2. Recruitment and consent

Patients will be recruited from the outpatient clinic of the department of Internal Medicine of the Ziekenhuisgroep Twente. Prior to their visit to the outpatient clinic, patients will be invited to participate in the study by sending a letter. In this letter, patients will find a full explanation of the study, advantages and disadvantages of participating, and contact information of the research team members working on this study. Moreover, the letter contains contact information of an independent physician, to whom subjects can address questions about the research before, during and after a study. The patients will be given 2 weeks to consider their decision and will then be asked to sign their written informed consent before they take part in the study.

11.3. Objection by minors or incapacitated subjects

No minors or incapacitated adults will be included in this study.

11.4. Benefits and risk assessment, group relatedness

The advantage of an N=1 study is that efficacy of the intervention is vetted for the actual participant. Since SGLT2 inhibitors are marketed in the Netherlands and recommended in patients with elevated albuminuria and type 2 diabetes mellitus, patients with diabetes who respond to dapagliflozin as determined by this study can continue to be treated with dapagliflozin. This does not yet apply to patients without diabetes. Participation in the study is on a free-will base. Patients will receive restitutions of all costs of transportation. Patients will not receive priority for treatment of other diseases in the clinic during this study. Participation in the proposed study is accompanied with only minor risks.

11.5. Compensation for injury

The investigator has a liability insurance which is in accordance with article 7 of the WMO.

The sponsor University Medical Center Groningen (UMCG) has an insurance which is in accordance with the legal requirements in the Netherlands (Article 7 WMO). This insurance provides cover for damage to research subjects through injury or death caused by the study. The insurance applies to the damage that becomes apparent during the study or within 4 years after the end of the study. All patients will receive written information about this insurance.

The sponsor/investigator has a liability insurance which is in accordance with article 7, subsection 6 of the WMO.

The sponsor (also) has an insurance which is in accordance with the legal requirements in the Netherlands (Article 7 WMO and the Measure regarding Compulsory Insurance for Clinical Research in Humans of 23th June 2003). This insurance provides cover for damage to research subjects through injury or death caused by the study.

1. € 650.000, - (i.e. four hundred and fifty thousand Euro) for death or injury for each subject who participates in the Research;
2. € 5.000.000, - (i.e. three million five hundred thousand Euro) for death or injury for all subjects who participate in the Research;
3. € 7.500.000, - (i.e. five million Euro) for the total damage incurred by the organisation for all damage disclosed by scientific research for the Sponsor as ‘verrichter’ in the meaning of said Act in each year of insurance coverage.

The insurance applies to the damage that becomes apparent during the study or within 4 years after the end of the study.

11.6. Incentives

Participation of patients in the study is a free-will decision. Patients will receive restitution of all costs for transportation. Patients do not receive priority for treatment of other diseases in the clinic during this trial.

12. ADMINISTRATIVE ASPECTS, MONITORING AND PUBLICATION

12.1. Handling and storage of data and documents

A subject identification code list will be made to link the data to the subject in order to be able to trace data to an individual subject. This code will not be based on the patient initials and birthdate. The key to the code will be safeguarded by the investigator since the data will be kept for a period of 15 years. The handling of personal data will comply with the General Data Protection Regulation (GDPR) (in Dutch: De Algemene verordening gegevensbescherming, AVG).

12.2. Monitoring and Quality Assurance

Independent clinical site monitoring and quality assurance is conducted at ZGT hospital to ensure that the rights and well-being of human subjects are projected, that the reported trial data are accurate, complete, and verifiable, and that the conduct of the trial is in compliance with the currently approved protocol, with GCP, and with applicable regulatory requirement(s).

12.3. Amendments

A ‘substantial amendment’ is defined as an amendment to the terms of the METC application, or to the protocol or any other supporting documentation, that is likely to affect to a significant degree:

- the safety or physical or mental integrity of the subjects of the trial;
- the scientific value of the trial;
- the conduct or management of the trial; or
- the quality or safety of any intervention used in the trial.

All substantial amendments will be notified to the METC and to the competent authority.

Non-substantial amendments will not be notified to the accredited METC and the competent authority but will be recorded and filed by the sponsor.

12.4. Annual progress report

The sponsor/investigator will submit a summary of the progress of the trial to the accredited METC once a year. Information will be provided on the date of inclusion of the first subject, numbers of subjects included and numbers of subjects that have completed the trial, serious adverse events/ serious adverse reactions, other problems, and amendments.

12.5. Temporary halt and (prematurely) end of study report

The sponsor will notify the accredited METC and the competent authority of the end of the study within a period of 90 days. The end of the study is defined as the last patient's last visit.

The sponsor will notify the METC immediately of a temporary halt of the study, including the reason of such an action.

In case the study is ended prematurely, the sponsor will notify the accredited METC and the competent authority within 15 days, including the reasons for the premature termination.

Within one year after the end of the study, the investigator/sponsor will submit a final study report with the results of the study, including any publications/abstracts of the study, to the accredited METC and the Competent Authority.

12.6. Public disclosure and publication policy

Publication policy is in agreement with the CCMO publication statement. Nor the sponsors, nor the principal investigator has a right of veto regarding the way of publishing the results.

13. STRUCTURED RISK ANALYSIS

13.1. Potential issues of concern

a. Level of knowledge about mechanism of action

The sodium glucose co-transporter 2 (SGLT2), located in the proximal tubule of the kidney, is an effective transporter system which is responsible for the nearly complete reabsorption of glucose in order to maintain appropriate glucose levels. Each glucose molecule that is reabsorbed is accompanied by reabsorption of a sodium molecule in a 1:1 ratio. SGLT2 inhibitors reversely inhibit the SGLT2 transporter which leads to enhanced glucose and sodium excretion and a reduction in plasma glucose and HbA1c. The effects of dapagliflozin on the SGLT2 transporter are well characterized and sufficient knowledge is available about the mechanisms of action.

b. Previous exposure of human being with the test product(s) and/or products with a similar biological mechanism

Previously, twelve phase 3 randomized controlled clinical trials were conducted involving more than 6000 patients with type 2 diabetes mellitus of whom ~4000 were treated with dapagliflozin. Eleven studies were 24-weeks in duration with extension in 6 studies up to 78 weeks. One study was 52 weeks in duration with extension of another 52 weeks.

More recently, the DAPA-CKD trial reported the efficacy and safety of dapagliflozin in 4,304 patients with chronic kidney disease with or without type 2 diabetes. The trial demonstrated that dapagliflozin significantly reduced the risk of kidney and cardiovascular endpoints and prolonged survival. Dapagliflozin was well tolerated in keeping with the established safety profile. Specifically, in this population there was no diabetic ketoacidosis with dapagliflozin. In patients with CKD without type 2 diabetes no major hypoglycemic events were reported.

c. Can the primary or secondary mechanism be induced in animals and/or in ex-vivo human cell material?

Various animal models and cell lines are available to study the effect of SFGLT2

inhibitors in more detail at a tissue/cell level.

d. Selectivity of the mechanisms to target tissue in animals and/or human beings

e. Analysis of potential effect

In placebo controlled clinical trials the following adverse reactions have been identified:

- Infections and infestations: vulvovaginitis, balanitis and related genital infections, urinary tract infections (common); fungal infection (uncommon)
- Metabolism and nutrition disorders: hypoglycemia (when used with SU or insulin) (very common); volume depletion (uncommon)
- Nervous system disorders: dizziness (common)
- Gastrointestinal disorders: constipation, dry mouth (uncommon)
- Skin and subcutaneous tissue disorders: rash (common)
- Musculoskeletal and connective tissue disorders: back pain (common)
- Renal and urinary disorders: dysuria, polyuria (common); nocturia (uncommon)
- Reproductive system and breast disorders: vulvovaginal pruritis, pruritus genital (uncommon)
- Investigations: hematocrit increased, creatinine renal clearance decreased during initial treatment, dyslipidemia (common); blood creatinine increased during initial treatment, blood urea increased, weight decreased (uncommon).

Common (>1/100 to <1/10) and uncommon (>1/1000 to <1/100)

Few adverse events led to discontinuation of treatment and adverse events were balanced across study groups. The most commonly reported events leading to discontinuation in patients treated with dapagliflozin 10 mg/day were increased blood creatinine (0.4%), urinary tract infections (0.3%), nausea (0.2%), and rash (0.2%). It should be noted that the transient rise in serum creatinine may reflect a reduction in intra-glomerular pressure which may be associated with long-term structural renal protection.

A pooled analysis of 3152 patients who received dapagliflozin in doses between 2.5 and 10 mg/day showed that the incidence of urinary tract infection was increased with dapagliflozin dosage. Most diagnosed infections were mild to moderate and responded to standard antimicrobial treatment.[17]

Overdose:
Dapagliflozin did not show any toxicity in healthy subjects at single oral doses up to 500 mg (50 times the dose aimed to be used in the present study). These subjects had detectable glucose in the urine for a dose-related period of time (at least 5 days for the 500 mg dose) with no reports of dehydration, hypotension, or electrolyte imbalance and with no clinically meaningful effect on QT_c interval. The incidence of hypoglycemia was similar to placebo. In clinical studies where once-daily doses of up to 100 mg dapagliflozin were administered for 2 weeks in healthy subjects and type 2 diabetes mellitus subjects, the incidence of hypoglycemia was slightly higher than placebo and was not dose-related. Rates of adverse events including dehydration or hypotension were similar to placebo, and there were no clinically meaningful dose-related changes in laboratory parameters, including serum electrolytes and biomarkers of renal function. In the event of an overdose, appropriate supportive treatment should be initiated as dictated by the patient's clinical status. The removal of dapagliflozin by hemodialysis has not been studied.

f. Pharmacokinetic considerations
In in-vitro studies, dapagliflozin neither inhibited cytochrome P450 (CYP) 1A2, CYP2A6, CYP2B6, CYP2C8, CYP2C9, CYP2C19, CYP2D6, CYP3A4 nor induced CYP1A2, CYP2B6, CYP3A4. Therefore, dapagliflozin is not expected to alter the metabolic clearance of co-administered medicinal products that are metabolized by these enzymes.

Interaction studies conducted in healthy subjects, using mainly a single dose design, suggest that the pharmacokinetics of dapagliflozin are not altered by metformin, pioglitazone, sitagliptin, glimepiride, voglibose, hydrochlorothiazide, bumetanide, valsartan, or simvastatin.

g. Study population

The enrolled population is in a stable condition and no unexpected serious adverse events are foreseen.

h. Interactions with other products

See F

i. Predictability of effect

Efficacy is monitored by measuring the urine albumin-to-creatinine ratio / blood pressure / body weight / eGFR / plasma glucose, which are accepted and accurate surrogates to evaluate efficacy.

j. Can effects be managed?

Patients are regularly monitored and asked about adverse effects of urinary tract infections or infestations. As reported above, dapagliflozin administration is associated with an increased risk of infections which can be managed with standard antimicrobial treatment.[16]

13.2. Synthesis

The available data show that dapagliflozin decreases HbA1c, blood pressure and body weight in patients with type 2 diabetes. The drug received marketing approval from the EMA and is registered in various EU-countries. Dapagliflozin increases incidence of urinary tract infections. This adverse effect led in rare instances to treatment discontinuation and is manageable with standard antimicrobial treatment.

14. REFERENCES

1. Bakris GL, Fonseca VA, Sharma K, et al. Renal sodium-glucose transport: role in diabetes mellitus and potential clinical implications. *Kidney Int.* Jun 2009; 75(12): 1272-1277.
2. Bae JH, Park E, Kim S, Kim SG, Hahn S, Kim NH. Effects of Sodium-Glucose Cotransporter 2 Inhibitors on Renal Outcomes in Patients with Type 2 Diabetes: A Systematic Review and Meta-Analysis of Randomized Controlled Trials. *Nature*. Sep 2019; 9(1): 13009.
3. Beitelshees AL, Leslie BR, Taylor SI. Sodium-Glucose Cotransporter 2 Inhibitors: A Case Study in Translational Research. *Diabetes.* Jun 2019; 68(6): 1109-1120.
4. Zannad F, Ferreira JP, Pockock SJ, Anker SD, Butler J, Filippatos G, et al. SGLT2 inhibitors in patients with heart failure with reduced ejection fraction: a meta-analysis of the EMPEROR-Reduced and DAPA-HF trials. *Lancet*. Sep 2020; 396(10254): 819-829.
5. American Diabetes Association. Microvascular Complications and Foot Care. *Diabetes Care*. Jan 2015; 38(1): S58-S66.
6. Bailey CJ, Gross JL, Pieters A, Bastien A, List JF. Effect of dapagliflozin in patients with type 2 diabetes who have inadequate glycaemic control with metformin: a randomized, double-blind, placebo-controlled trial. *Lancet.* Jun 2010; 26: 375(9733): 2223-33.
7. Dhillon S. Dapagliflozin: A Review in Type 2 Diabetes. *Drugs.* Jul 2019; 79(10): 1135-1146.
8. Lambers Heerspink HJ, Stefánsson BV, Correa-Rotter R, Chertow GM, Greene T, Hou F, et al. Dapagliflozin in Patients with Chronic Kidney Disease. *N Eng J Med*. Oct 2020; 383(15): 1436-1446.
9. Petrykiv SI, de Zeeuw D, Persson F, Rossing P, Gansevoort RT, Laverman GD, Lambers Heerspink HJ. Variability in response to albuminuria-lowering drugs: true or random? *Br J Clin Pharmacol.* 2017 Jun; 83(6): 1197-1204.
10. Petrykiv SI, Laverman GD, de Zeeuw D, Lambers Heerspink HJ. The albuminuria-lowering response to dapagliflozin is variable and reproducible among individual patients. *Diabetes Obes Metab.* Oct 2017; 19(10): 1363-1370.
11. Perkovic V, Jardine MJ, Neal B, Bompoint S, Lambers Heerspink HJ, Charytan

DM, et al. Canagliflozin and Renal Outcomes in Type 2 Diabetes and Nephropathy. *N Eng J Med.* Jun 2019; 380(24): 2295-2306.

12. Wiviott SD, Raz I, Bonaca MP, Mosenzon O, Kato ET, Cahn A, et al. Dapagliflozin and Cardiovascular Outcomes in Type 2 Diabetes. *N Eng J Med.* Jan 2019; 380(4): 347-357.
13. British Hypertension Society. Home Blood Pressure Monitoring Protocol. [Internet]. Available from: https://bihsoc.org/wp-content/uploads/2017/09/Protocol.pdf. [Accessed 29th of September)
14. George J, MacDonald T. Home Blood Pressure Monitoring. *Eur Cardiol.* Dec 2015; 10(2): 95-101.
15. Sharman JE, Howes F, Head GA, McGrath BP, Stowasser M, Schlaich M. How to measure home blood pressure: Recommendations for healthcare professionals and patients. *Aust Fam Physician.* Jan-Feb 2016; 45(1): 31-4.
16. Johnsson KM, Ptaszynska A, Schmitz B, et al. Urinary tract infections in patients with diabetes treated with dapagliflozin. *J Diabetes Complications.* Sep-Oct 2013; 27(5): 473-478.

The @home n =1 trial

Individual albuminuria lowering response to dapagliflozin in a decentralized clinical trial in patients with type 2 diabetes mellitus and elevated albuminuria

STATISTICAL ANALYSIS PLAN

Version 1.0
25 October 2022

Signature Page

Titel study: Individual albuminuria lowering response to dapagliflozin in a decentralized clinical trial in patients with type 2 diabetes mellitus and elevated albuminuria

Investigator:

Hiddo L Heerspink Date 25 October 2022

1. Study synopsis

Persistent increased albuminuria is a strong risk marker for progressive kidney disease and cardiovascular disease in patients with or without diabetes. The degree of albuminuria reduction in the first months of treatment with pharmacological or dietary intervention correlates with the degree of long-term (3 to 4 years) renal or cardiovascular protection. Despite the various available treatments to decrease urinary albumin excretion, residual albuminuria persists in many patients. The high residual albuminuria in a proportion of patients is at least in part explained by suboptimal response to the current treatments (i.e., ACE inhibitor or Angiotensin Receptor Blockers).

Dapagliflozin is a sodium-glucose transport inhibitor and inhibits the reabsorption of glucose in the proximal tubule. This leads to a decrease in fasting plasma glucose and HbA1c in patients with type 2 diabetes. In addition, dapagliflozin administration causes a decrease in blood pressure and body weight and an increase in haematocrit suggestive of a diuretic effect. Previous studies have also demonstrated the albuminuria lowering effects of dapagliflozin in patients with type 2 diabetes mellitus.

Although dapagliflozin markedly slows progression of kidney function decline (and reduces cardiovascular outcomes) on a population level, randomized parallel group trials have suggested a marked variation in the response to dapagliflozin between individual patients. By design, randomized parallel group placebo-controlled clinical trials test the efficacy of new interventions on a population level but do not assess the efficacy of a drug for the individual. Although there is variation in response between patients, parallel group trial does not allow conclusions whether this variation is a true variation in drug response, or measurement or temporal random variation. We therefore propose a cross-over trial with repeated administration (i.e., a series of N=1 trials) to ascertain the individual drug response. This design

2. Study objectives

Our aim is to investigate the individual response in urinary albumin-to-creatinien ratio to the Dapagliflozin 10 mg/day.

2.1. Primary Objective

- To determine the individual response to the SGTL2 inhibitor dapagliflozin in urine albumin-to-creatinine ratio (UACR)

2.2. Secondary Objectives

- To determine the individual response to the SGLT2 inhibitor dapagliflozin in:
  - Systolic blood pressure
  - Body weight
  - eGFR
  - Fasting plasma glucose

3. Study design

The trial will be performed in the remote setting. In a conventional clinical trial, patients have to visit the hospital/clinic multiple times for health check-ups and blood and / or urine collections. This may complicate the feasibility of the trial since not all patients are willing to frequently visit the clinic, which hampers recruitment. In addition, patients may drop out during the trial since the time investment and burden of study visits is higher than originally expected. With the introduction of digital technologies and wearable devices, it becomes possible to assess patients remotely – while they are at home. If used in a clinical trial, it could significantly reduce the number of study visits, time investment for patients, and increase the operational processes of clinical trial conduct.

Some healthcare providers and IT suppliers already offer patients the opportunity to create and manage a personal health record at a central privacy secured server. More and more patients are also keeping their own records, but the scope for combining and sharing all these records is still somewhat limited. Based on a strong believe that people play a vital role in their own health, various parties in the healthcare sector have taken the initiative to set up special platforms to offer people more control over their own health. In this study, we will use such an online platform (Selfcare). Selfcare creates a personal health environment (PHE), a digital tool in which patients can store all their health data in a safe, privacy secured, and independent manner. Up to now, health data has not always been easily accessible, but this PHE allows patients to safely compile and share their own health information online with physicians and researchers.

To take advantage of these developments we will perform this study using digital technologies and ask patients to collect study data at home.

3.1. General Design and Plan

Subjects with UACR >20 mg/g (2.26 mg/mmol) with a diagnosis of type 2 diabetes who meet the inclusion criteria will be invited for screening. After the screening visit, eligible patients will proceed to the randomization visit. Patients will be randomly assigned to a cross-over study consisting of two periods of 1-week treatment with dapagliflozin and two periods of 1-week treatment with placebo in random order with a 1-week wash-out period between every treatment period to avoid cross-over effects. Based on a prior study, effects of dapagliflozin on UACR, blood pressure, body weight, fasting plasma glucose and eGFR were fully present after 4 days. We will therefore ask patients to collect urine after 3, 5, 6, and 7 days. Due to day-to-day variation in UACR we are collecting multiple urine samples to increase precision and therefore collect urine at 3, 5, 6, and 7 days. We will also collect urine after 1 day to further delineate the time-course of the response to dapagliflozin and assess if the treatment effect on UACR is directly present after 1 day. Blood pressure and body weight will be recorded, and blood samples will be taken at the time points described below and in the table. Overall, a 1-week treatment period is considered sufficient to detect effects on primary and secondary outcomes.

Since data collection will be performed at home using wearable devices and digital technologies, instructions will be provided during the randomization visit to perform data collection and study procedures at home. Data collection and measurements will be performed in each treatment period as indicated in Figure 1. Bottles with smart caps that

allow real-time monitoring of adherence, blood pressure monitors, body analysis scales and study medication will be provided at the randomization visit. Patients receive four numbered bottles with seven tablets in each bottle, according to their randomized treatment schema. Patients will be instructed to take the study medication once daily, in the morning.

Measurements:

- First morning void urine collections for measurement of UACR will be performed at:
  - Day -7, -3, -2 and -1
  - Day 1, 3, 5, 6 and 7 of the active periods
  - Day 3, 5, 6 and 7 of the wash-out periods
- Ambulatory blood pressure will. Be measured by the patients themselves at home at:
  - Day -7, -3, -2, and -1
  - Day 1, 3, 6 and 7 of the active periods
  - Day 6 and 7 of the wash-out periods
- Ambulatory body weight will be measured by the patients themselves at home at:
  - Day -7, -3, -2, and -1
  - Day 1, 3, 6 and 7 of the active periods
  - Day 6 and 7 of the wash-out periods
- Capillary blood samples for measurement of creatinine, CRP, lipid profile, HbA1c, uric acid, glucose and NT-proBNP will be taken at:
  - Day -7 (under supervision during the randomization visit) and -1
  - Day 1, 6 and 7 of the active periods
  - Day 6 and 7 of the wash-out periods

A venous blood sample will also be taken during the randomization visit in order to compare the clinical chemistry assessments in capillary blood with those measured in venous blood samples.

- Medication will be provided in bottles with smart caps that allow real-time monitoring of adherence.
- Dapagliflozin will be measured by a validated liquid chromatography-mass spectrometry (LC-MS/MS) analysis at day 7 of every active period in capillary blood samples to assess treatment adherence.
- Telephone calls will be made at day 3 and 6 of the first two treatment periods to assess compliance with study procedures.

Questionnaires consisting of 6 multiple choice questions and 2 open questions will be used to assess patient experience and satisfaction to guide and inform future trials and use in clinical practice.

Figure 1: Study design

					Period 1					Wash-out				Period 2/3/4					Wash-out			
**Days**	-7	-3	-2	-1	1	3	5	6	7	3	5	6	7	1	3	5	6	7	3	5	6	7

**UACR (PeeSpot)**	X	X	X	X	X	X	X	X	X	X	X	X	X	X	X	X	X	X	X	X	X	X
**Blood pressure**	X	X	X	X	X	X		X	X			X	X	X	X		X	X			X	X
**Body weight**	X	X	X	X	X	X	X	X	X	X	X	X	X	X	X	X	X	X	X	X	X	X
**Blood-samples* (Hem-Col)**	X			X	X			X	X			X	X	X			X	X			X	X
**Glucose (strips)**	X			X	X			X	X			X	X	X			X	X			X	X
**Treatment adherence (MEMS)**	X	X	X	X	X	X	X	X	X	X	X	X	X	X	X	X	X	X	X	X	X	X
**Dapagliflozin concentration (LC-MS/MS)**									X									X				
**Questionnaire**																						X

*Creatinine, CRP, lipid profile, HbA1c, uric acid and NT-proBNP will be measured

Patients connect their blood pressure monitor and body analysis scale to the corresponding mobile application, which in turn is linked to Selfcare platform. In this way, data will be automatically transferred to Selfcare platform. Patients can also register adverse events in Selfcare. Selfcare creates a personal health environment (PHE), a digital tool in which patients can store all their health data in a safe and independent manner. These data are integrated on a single independent platform. This unique dynamic dashboard forms a reliable basis on which health data can be monitored. At the end of the study, blood pressure and body weight data will be downloaded from Selfcare and along with the biochemical data measured in the central laboratory incorporated in the database.

Selfcare provides the best possible data protection. Selfcare meets the Dutch standard for information security in the healthcare sector (NEN 7510), which relates to the formulation, registration and monitoring of information security. This standard entails: ‘Ensuring the availability, integrity and confidentiality of all information needed to provide patients with responsible care.’

Selfcare users have full ownership of their personal health data at all times. Only if the user gives explicit consent, third parties can be granted access to the data. Communication with peers in the in the community and data exchange (if permitted) takes place in a secure environment. Selfcare guarantees privacy whether a patient uses a computer, tablet or smartphone.

When users open Selfcare dashboard from a new device, they are asked to provide a second piece of information (in addition to their username and password) to prove their identity. This is an additional step taken by Selfcare to prevent abuse and provide personal safety.

Selfcare uses HTTPS, a protocol that is used for the secure handling of request between a client (browser) and server (web server). Data are encrypted, making it virtually

impossible for someone without proper authorization to access the data. The selfcare servers are hosted at a national level by independent foundations to ensure privacy.

The Selfcare data are subjected to independent supervision by leading authorities in the field of privacy and patient protection. All personal user data are stored on a server, which is located in the country of registration. Ownership of this server lies with a legal entity that is separated from the commercial organization Selfcare. This means that the data are never accessible to third parties and can never be sold to third parties. This ensures that only the end user determines what happens to his or her data.

3.2. Sample Size

The sample size for this study is based on the hypothesis that the response to dapagliflozin is reproducible upon re-exposure and thus correlates between the first and second exposure while no correlation will be detected during placebo patients. A prior retrospective study found that during intervention in the renin-angiotensin-system responses in proteinuria correlate with correlation coefficients ranging between 0.7 to 0.9. Since this was a retrospective study, we assume a more conservative correlation coefficient of 0.6. A sample size of 20 patients provides >80% power (alpha 0.05) to detect a correlation coefficient of 0.6.

4. Study population

The study population will consist of adult male and female patients with UACR [illegible]20 mg/g (2.26 mg/mmol) with T2DM treated in primary or secondary healthcare. Subjects will be recruited via general practitioner practices or via the outpatient clinic of the Department of Internal Medicine of the Ziekenhuisgroep Twente, Almelo.

<u>Inclusion criteria</u>

- Age >18 years
- Diagnosis of type 2 diabetes mellitus
- Urinary albumin-to-creatinine ratio >20 mg/g (2.26 mg/mmol)
- eGFR >30 ml/min/1.73m2
- Willing to sign informed consent

<u>Exclusion criteria.</u>

- Diagnosis of type 1 diabetes.
- Prior treatment with SGLT2 inhibitor in the four weeks prior to randomization.
- History of severe hypersensitivity or contraindications to dapagliflozin.
- Unable to monitor blood pressure / body weight or handle digital technologies.
- History of non-adherence to medical regimens or unwillingness to comply with the study protocol.
- Participation in any clinical investigation within 3 months prior to initial dosing.
- Unstable or rapidly progressing renal disease.

- Severe hepatic impairment (Child-Pugh class C) as determined by the treating physician.
- Active malignancy.
- Any medication, surgical or medical condition which might significantly alter the absorption, distribution, metabolism, or excretion of medications including, but not

limited to any of the following: o History of active inflammatory bowel disease, within the last six months.
  - Major gastrointestinal tract surgery as decided by the treating physician
  - Pancreatitis within the last six months.
  - Evidence of serious hepatic disease as determined by the treating physician
  - Evidence of urinary obstruction or difficulty in voiding at screening.
- Confirmed lactose intolerance demonstrated with a lactose intolerance test.
- Donation or loss of 400 mL of blood within 8 weeks prior to initial dosing.
- History of drug or alcohol abuse within the 12 months prior to dosing, or evidence of such abuse as indicated by the laboratory assays conducted during the screening
- Any surgical or medical condition, which in the opinion of the investigator, may place the patient at higher risk from his/her participation in the study, or is likely to prevent the patient from complying with the requirements of the study or completing the study.
- Current pregnancy or breast feeding / attempting to conceive.
- Women of childbearing potential (WOCBP):
  - WOCBP who are unwilling or unable to use an acceptable method of contraception to avoid pregnancy throughout the study and for up to 4 weeks after the last dose of study drug in such a manner the risk of pregnancy is minimized.
  - WOCBP must have a negative serum or urine pregnancy test result (minimum sensitivity 25 IU/L or equivalent of HCG) within 0 to 72 hours before the first dose of study drug.

WOCBP comprises women who have experienced menarche and who have not undergone successful surgical sterilization (hysterectomy, bilateral tubal ligation, or bilateral oophorectomy) or who are not post-menopausal (see definition below). The following women are WOCBP:

- Women using the following methods to prevent pregnancy: oral contraceptives, other hormonal contraceptives (vaginal products, skin patches, or implanted or injectable products), or mechanical products such as intrauterine devices or barrier methods (diaphragm, condoms, spermicides).
- Women who are practicing abstinence.
- Women who have a partner who is sterile (e.g. due to vasectomy).

Post-menopause is defined as:

- Women who have had amenorrhea for >12 consecutive months (without another cause) and who have a documented serum follicle-stimulating hormone (FSH) level >35 mIU/mL.
- Women who have irregular menstrual periods and a documented serum FSH level >35 mIU/mL.
- Women who are taking hormone replacement therapy (HRT).

4.1. Procedures

Recruitment:
Patients will be recruited from the outpatient clinic of the department of Internal Medicine of the Ziekenhuisgroep Twente. Prior to their visit to the outpatient clinic, patients will be invited to participate in the study by sending a letter.

Randomization and treatment allocation:
Treatment sequence of dapagliflozin and placebo will be randomized by the pharmacy unit of the University Medical Center Groningen. A computer-generated randomized code will be used. The pharmacy of the University Medical Center Groningen will store the randomization code.

Measurements:
Data collection will be performed at home using wearable devices and digital technologies. Data collection and measurements will be performed in each treatment period as indicated in Figure 1. The following devices will be used to measure bio-physical/anthropomorphic variables:

- *Body weight measurements:*
  Body weight will be measured by the Withings® Body+ (Withings, Issy-les Moulineux, France) device, which is a clinically validated advanced Wi-Fi smart scale that performs highly accurate bioelectrical impedance analysis (weight, body fat & water percentage, plus muscle & bone mass). Patients will be instructed to always weigh themselves under identical conditions. Preferably, weighing will take place in the morning, after showering or bathing, and with dry feet, but before breakfast. Patients will also be instructed to stand up straight and stand still.

- *Blood pressure measurements:*
  The Withings® BPM Connect device will be used to monitor blood pressure. This clinically validated Wi-Fi blood pressure monitor provides medically accurate blood pressure and heart rate measurements compliant with European medical device standards. The results are within the margin of acceptance defined by the internationally recognized evaluation standard of blood pressure monitors ANSI/AAMI/ISO 81060-2:2013, EN ISO 81060-2:2014, developed by the European Society of Hypertension, British Hypertension Society and Association for the Advancement of Medical Instrumentation/American Heart Association. Both of the Withings devices will be connected with a smartphone app in order to transfer the data to Selfcare platform. Prior to starting home blood pressure monitoring, patients will be instructed to measure their blood pressure in both arms to determine which arm should be used for future measurements. The arm that gives the higher systolic reading should be used for all future testing. Patients should place the cuff on their arm with the lower edge of the cuff approximately 2cm above the bend in their elbow. The center of the inflatable bladder should be positioned over the brachial artery on the arm's inferior surface. The forearm should be supported on a firm surface and should be at the level of the lower end of the breastbone. No tight or

restrictive clothing should be worn around the arm. Patients should be seated, remain silent and be at rest for a minimum of five minutes before taking a measurement and should not have smoked, eaten, drunk a caffeinated drink or undertaken physical activity within the past thirty minutes. Patients should also avoid measuring their blood pressure with. a full bladder. Measurements should be taken in silence when the patient is relaxed, with both feet flat on the floor and their back and arm supported. Many patients automatically cross their legs, which raises their blood pressure, so it is particularly important to emphasize the need for the patient to uncross their legs when taking their blood pressure. For each BP recording, three consecutive measurements are taken, at least 1 minute apart with the person seated. BP is recorded once daily.

Blood and urine will be sampled with dedicated devices:

- *Blood sampling:*
  Capillary blood will be sampled using a BD Microtainer® Contact-Activated Lancet (Franklin Lakes, New Jersey, USA). Blood is collected with the Hem-Col® device, which is designed to collect capillary blood drawn with a finger prick. Hem-Col is a microtube containing an anticoagulant and a preservation buffer to enhance analyte stability in whole blood. The Hem-Col® device containing 17 USP/mL lithium heparin dissolved in 150 µL preservation fluid (Hem-Col, Labonovum, Limmen, the Netherlands) will be used. Hem-Col tubes have the size of regular blood collection tubes (13 x 75 mm) and are made of polyethylene, with a pierceable cap made of thermoplastic elastomers. All tubes contain a liquid barrier, the inner part serves as a liquid barrier by preventing loss of Hem-Col conservation fluid and the outer part is used as a scoop to collect blood from a finger prick. Hem-Col lithium heparin will be used for analyses of creatinine, C-reactive protein (CRP), lipid profile, HbA1c, uric acid and the N-terminal prohormone of brain natriuretic peptide (NT-proBNP). The Hem-Col device can be sent to the laboratory for analysis by regular post. Capillary blood sampled by BD Microtainer® Contact-Activated Lancet will also be used to determine fasting plasma glucose. If a patient already has an eligible glucose meter, this device can be used in order to determine fasting plasma glucose. In this case, patients have to manually enter their blood glucose results in Selfcare platform. If a patient does not already have a glucose meter, an Accu-Chek® Instant System (Roche Diagnostics, GmbH, Mannheim, Germany) device with appropriate test strips (Accu-Chek® Instant) will be provided during the randomization visit. The accuracy of the Accu-Chek® Instant System is guaranteed by the makers of the Accu-Chek products, fulfilling the requirements of the ISO 15197:2013/EN ISO 15197:2015. Once connected to Google fit, the meter automatically logs blood glucose results and wirelessly transfers them to Selfcare platform.

- *First morning void urine collection:*
  Urine will be collected with the PeeSpot® (Hessels+Grob, Apeldoorn, the Netherlands) device, which is a validated tool for collection and conservation of small amounts of urine. It consists of a urine absorption pad, a holder, a tube and a

lid. Patients can void over the absorption pad while placed in the holder. After voiding, the pad and holder are placed in the tube, closed with the lid and kept in the refrigerator until sending to the laboratory. By adding an inert hygroscopic polymer, the pad easily absorbs 1.2 mL urine and by adding various preservatives, urine is preservable for 4 days. After completing the urine collections, the PeeSpots are placed in a safety bag and sent to the central laboratory in an envelope for biological materials (PolyMed, DaklaPack, Europe). We will use the PeeSpot to determine the first morning void UACR. The PeeSpot has been validated for measurement of urinary albumin and creatinine.

- *Treatment adherence:*
  Medication will be provided in standard medicine bottles with the MEMS® (Medication Electronic Monitoring System) Cap (AARDEX Ltd, Union City, CA, USA), which is a customizable medication package which records and stores up to 4,000 dosing events. The cap fits on standard medicine bottles and with integrated microcircuits, the child-resistant MEMS® Cap records the date and time whenever a patient opens a vial. The stored information can be transferred at any time through the MEMS® Reader to the adherence software for immediate analysis and interpretation.

- *Laboratory measurements:*
  Dapagliflozin concentration measurements will be performed at the University Medical Center Groningen using liquid chromatography mass-spectrometry (LC-MS/MS) in capillary blood samples. First morning void UACR measurements will also be performed at the laboratory of the University Medical Center Groningen. Blood (creatinine, CRP, lipid profile, HbA1c, uric acid and NT-proBNP) measurements will be performed at the laboratory (Medlon BV) of the Ziekenhuisgroep Twente. Residual urine and blood plasma samples will be stored at the University Medical Center Groningen in the context of this study to allow for future exploratory biomarker analyses to further study the effects of dapagliflozin on type 2 diabetes, albuminuria and kidney function. Samples will be stored with the same patient number used in the study database. The samples are only accessible to the principal investigator and other delegated members of the study team. No biobank is made.

5. Statistical analysis

Patients will be described with their demographical and medical data. Baseline characteristics will be presented as mean and standard deviation for parametric data and as median and 25th and 75th percentile for non-parametric data. Non-parametric data including 24-hour albuminuria and urinary albumin:creatinine ratio will be log-transformed for regression analyses and the reverse of the log will be presented in summaries and results.

5.1. Analysis of primary and secondary study outcome

The change from baseline in UACR will be calculated using the values collected at day 5, 6 and 7 of each treatment and wash-out period. Because of the skewed distribution UACR will be log-transformed before analysis. The geometric mean of these 3 values (at baseline to end of treatment) will be calculated as log(UACRend/UACRbaseline) and the geometric mean change from baseline will be determined.

The study has two cycles of two treatment periods (dapagliflozin and placebo). For the calculation of response, the difference in log-transformed UACR change between dapagliflozin and placebo will be calculated during the first and second cycle. *Patient level Bayesian analysis:*

First, a Bayesian analysis will be conducted to calculate the posterior probability of dapagliflozin producing a clinically meaningful response on UACR within each individual patient relative to placebo for both treatment cycles combined. Thus for every patient, data of each cycle is combined and expressed in the form of a likelihood function with non-informative priors to calculate the posterior distribution. A clinically meaningful response on UACR is defined as the placebo subtracted percentage UACR change during the first and second cycle with different thresholds of 10%, 20% or 30% reduction in UACR. *Population level Bayesian hierarchical analysis:*

Subsequently, individual patient results on UACR will be combined to conduct a Bayesian hierarchical analysis to calculate the posterior probabilities of a clinically meaningful response at the population level (10%, 20%, and 30%). Therefore, responses on UACR from each patient will be aggregated into a sample mean and variance, assuming a normal distribution centred around the patient's true mean effect and variance. The Bayesian hierarchical model is defined as:

$$Y_{ij} \sim N(a_i + \beta_i X_{ij}, \sigma_i).$$

Were Y_{ij} is the placebo subtracted UACR response for the ith subject at time j and is described by x_{ij} ($x_{ij} = 1$ if treated with dapagliflozin; $= 0$ if placebo treated). The subject specific intercept and slope were modelled as $a_i \sim N(a_0, \tau_a^2)$ and $\beta_i \sim N(\beta_0, \tau_\beta^2)$ respectively. Again, we used non-informative prior for a_0, β_0, τ_a^2, τ_β^2, σ_i.

We will repeat this analysis for the secondary outcomes such as blood pressure, body weight, and several clinical chemistry laboratory parameters, Similar to UACR, we will estimate the posterior probability of a clinically meaningful response and the overall treatment effect. clinically meaningful response as defined as:

- Blood pressure: reduction of 5 mmHg

- Body weight: reduction of 3kg
- Hba1c: reduction of 0.5%
- EGFR: reduction of 10% and 3 ml/min.

Frequentist linear mixed effects analysis:

In addition to this Bayesian approach, we will also utilize a frequentist approach to determine the response on UACR during each period. To estimate this response a linear mixed effects model with random intercepts and random slopes for treatment will be used. Fixed effects include treatment sequence, and treatment cycle. Baseline log-transformed UACR may be added as covariate. Appropriate contrasts will be implemented to assess the placebo subtracted response during the first and second cycle and to correlate the placebo adjusted UACR responses to dapagliflozin (fitted on the log-scale) during the two treatment periods. The variance-covariance pattern of the mixed effects model is assumed to be unstructured. If the model does not converge less stringent models will be considered.

Heterogeneity treatment of effect analysis:

For those patients that completed both treatment cycles we will evaluate heterogeneity of treatment effects (HTE) across patients using a linear regression model with UACR response as the outcome. The model includes fixed effects for treatment and subject and a treatment-by-subject interaction, this allows each participant to have a different treatment effect. The statistical significance of patient-level HTE will be assessed by a likelihood test for the interaction term. In addition, we also fit a generalised linear mixed effect (LME) model with a random intercept and slope (for the treatment effect) to estimate the average treatment effect across all subjects. Fixed effects include treatment sequence, and treatment cycle in combination with an unstructured covariance structure.

Also we will use the linear mixed effects model as described above to determine if there is within individual variation in treatment response, we will fit a linear mixed model with correlated random intercepts and random slopes for treatment and include treatment period as a fixed effect. Subsequently, we will compare (using a maximum-likelihood ratio fit test) this model against a random intercept only model with treatment and treatment period as fixed effects.

5.2. Sub group analysis:

The primary and secondary outcomes will be analyzed in the following subgroups by adding a subgroup parameter to the Bayesian hierarchical model as described above:

1. Age (≥ or < 65 years or median)
2. Gender (Male / Female)
3. Urinary albumin:creatinine ratio (≥ or < 300 mg/g (33.4 mg/mmol) or median)
4. eGFR (≥ or < 60 ml/min/1.73m^2 or median)
5. Systolic blood pressure (≥ or < 130 mmHg or median)
6. Body mass index (≥ or < 25 kg/m^2 or median)

6. Evaluation of safety parameters

Counts and percentage per treatment arm will generally summarize all categorical indicators. A breakdown of SAEs stratified by fatal/non fatal status will be presented.
