# Supplementary material for: Albuminuria Responses to Dapagliflozin in Patients With Type 2 Diabetes: A Crossover Trial
Source: JAMA Netw Open. 2025 Mar 24;8(3):e251689. doi: 10.1001/jamanetworkopen.2025.1689 (PMC11934004; doi:10.1001/jamanetworkopen.2025.1689)
Supplement: Supplement 2. — eFigure 1. Changes in systolic blood pressure, body weight, and eGFR during treatment with dapagliflozin and placebo eFigure 2. Medication adherence per active treatment day eMethods. Questionnaire [file jamanetwopen-e251689-s002.pdf]

Supplementary Online Content

Beernink JM, Jongs N, Doelman CJA, Laverman GD, Heerspink HJL. Albuminuria responses to dapagliflozin in patients with type 2 diabetes: a crossover trial. *JAMA Netw Open*. 2025;8(3):e251540. doi:10.1001/jamanetworkopen.2025.1540

eFigure 1. Changes in systolic blood pressure, body weight, and eGFR during treatment with dapagliflozin and placebo

eFigure 2. Medication adherence per active treatment day

eMethods. Questionnaire

This supplementary material has been provided by the authors to give readers additional information about their work.

© 2025 Beernink JM et al. *JAMA Network Open*.

eFigure 1. Changes in systolic blood pressure, body weight, and eGFR during treatment with dapagliflozin and placebo

A: Systolic blood pressure; B: Body weight; C: eGFR

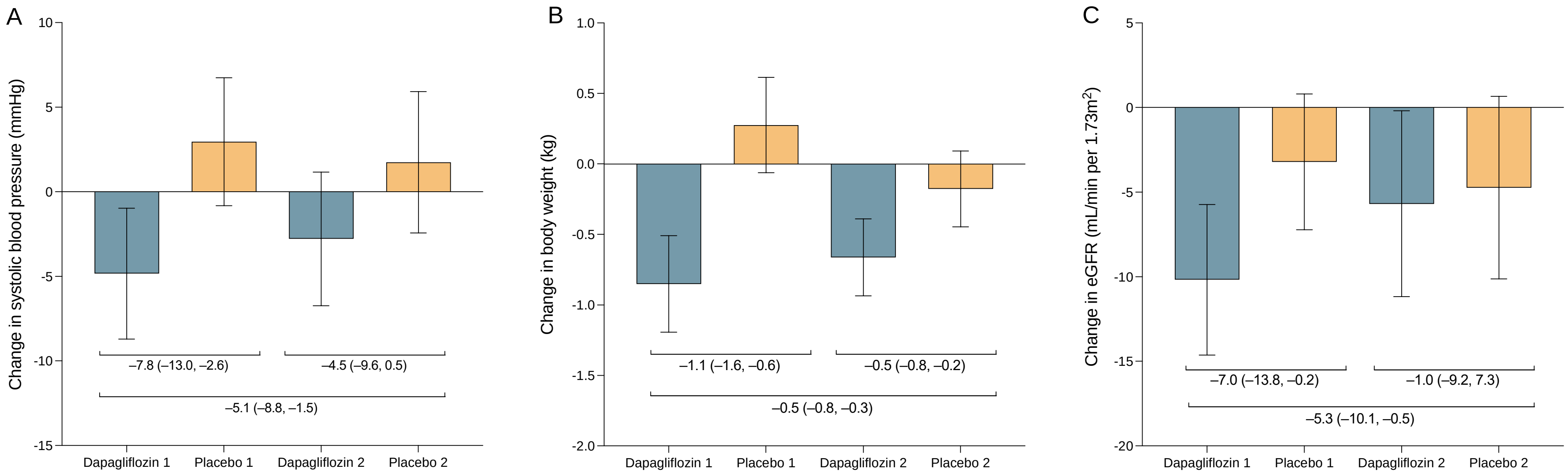

The figure shows the absolute changes from baseline. The error bars indicate the 95% CI.

© 2025 Beernink JM et al. *JAMA Network Open*.

eFigure 2. Medication adherence per active treatment day

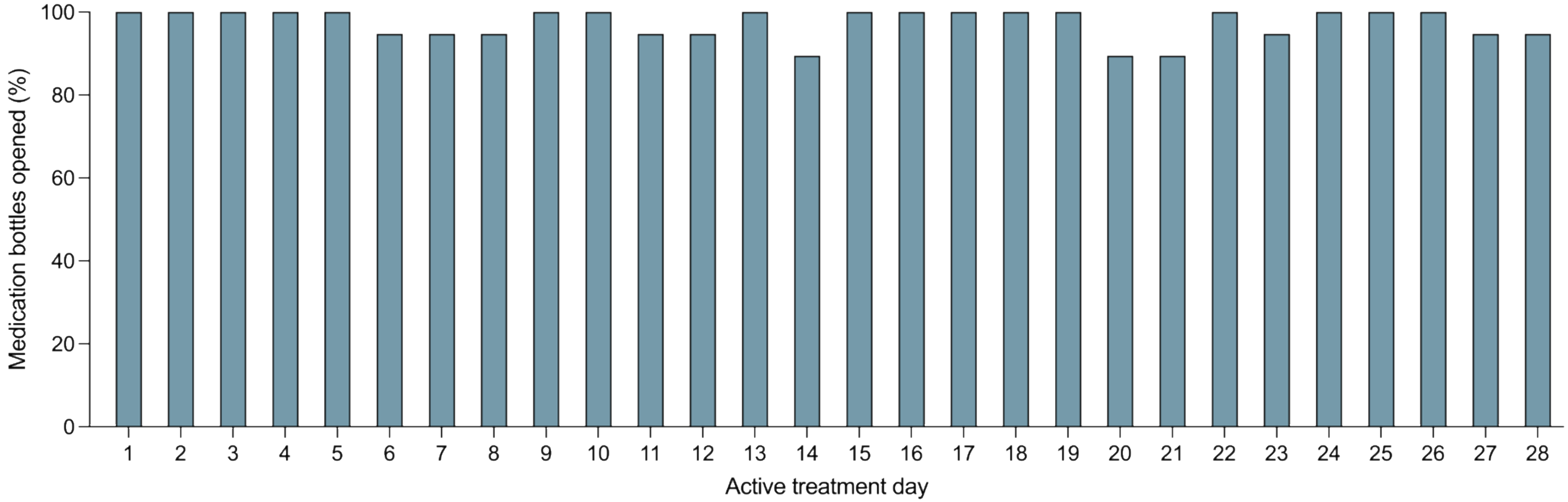

Percentage of medication bottles opened on the active treatment days. Medication adherence data for one participant is missing due to non-return of the medication bottles.

© 2025 Beernink JM et al. *JAMA Network Open*.

eMethods. Questionnaire

How did you experience the following tasks?
(Circle one number per line)

	Very difficult	Difficult	Neutral	Easy	Very easy
**1. Installing the mobile app(s)**	1	2	3	4	5
**2. Connecting measurement devices to the app(s)**	1	2	3	4	5
**3. Measuring blood pressure**	1	2	3	4	5
**4. Measuring weight**	1	2	3	4	5
**5. Collecting blood samples**	1	2	3	4	5
**6. Collecting urine samples**	1	2	3	4	5

7. Do you have any suggestions for improving the measurement procedures?
(Write your answer on the lines below)

………

………

………

………

8. Any additional feedback you can provide about this study is greatly appreciated and will help us in conducting future research.

© 2025 Beernink JM et al. *JAMA Network Open*.

(Write your answer on the lines below)

....

....

....

....

© 2025 Beernink JM et al. *JAMA Network Open*.
