# Supplementary material for: Albuminuria Responses to Dapagliflozin in Patients With Type 2 Diabetes: A Crossover Trial
Source: JAMA Netw Open. 2025 Mar 24;8(3):e251689. doi: 10.1001/jamanetworkopen.2025.1689 (PMC11934004; doi:10.1001/jamanetworkopen.2025.1689)
Supplement: Supplement 3. — Data Sharing Statement [file jamanetwopen-e251689-s003.pdf]

Data Sharing Statement

Beernink. Albuminuria Responses to Dapagliflozin in Patients With Type 2 Diabetes. *JAMA Netw Open*. Published March 24, 2025. doi:10.1001/jamanetworkopen.2025.1689

Data
Additional Information: EudraCT number: 2020-004929-23
Data available: Yes
Data types: Deidentified participant data
How to access data: Sponsor: University Medical Center Groningen
When available: With publication

Supporting Documents
Document types: None

Additional Information
Who can access the data: Researchers whose proposed use of the data has been approved.
Types of analyses: Specified purpose
Mechanisms of data availability: With investigator support, after approval of a proposal, with a signed data access agreement.
